# Intra-brand competition in a differentiated oligopoly

**DOI:** 10.1007/s00712-020-00712-w

**Published:** 2020-08-16

**Authors:** Michèle Breton, Lucia Sbragia

**Affiliations:** 1 Department of Decision Sciences, HEC Montréal, Montreal, Canada; 2 Department of Economics and Finance, Durham University Business School, Durham, UK

**Keywords:** Differentiated oligopoly, Cournot solution, Intra-brand competition, Equilibrium, C72, L13

## Abstract

In this paper we consider a differentiated oligopoly with two product varieties that are supplied by two groups of firms. After computing the Cournot solution of the game, we study its sensitivity to different sources of competition, namely the degree of product substitutability and market composition. Market composition can change either via new firms entering one industry or via firms switching production techniques, thus modifying the intensity of intra-brand competition. After studying the welfare consequences of an intensification of competition, we identify the equilibrium market composition when firms are driven by profit considerations. All the results are expressed in terms of the degree of product substitutability and of what we define “weighted relative efficiency” (WRE), which is a parameter combining both firm characteristics and market conditions.

## Introduction

In response to consumers’ increasing concern for the environment and interest in making greener choices,^1^ firms have started investing in production practices that allow them to receive a label certifying their compliance with certain set standards (e.g. organic, bio, sustainable, fair trade). Firms that earn a “green” label diversify their product offering from a conventional one. For example, in the fishery industry, when a company adopting a specific production technique receives the Marine Stewardship Council (MSC) label following a certification process, it diversifies its product offering and competes against companies selling non MSC-certified products. Moreover, certified firms may also compete against each other. In this paper, we use the term *inter-brand competition* to designate competition among firms selling different but interchangeable products (substitute goods), and *intra-brand competition* to designate competition among firms selling the same (homogenous) product.

Inter-brand and intra-brand competition have been mainly studied in the contexts of vertical agreements or of supply channels. In the legal literature, issues consist of assessing whether vertical agreements (e.g. between manufacturers and retailers) may prevent or restrict competition. In the economics literature, vertical agreements in oligopolistic markets have been analyzed under a strategic perspective, for instance to determine the equilibrium contract offered by the upstream firms and the resulting impact on downstream competition (see for instance Saggi and Vettas 2002), or to examine whether cooperation or collusion among competitors may be beneficial (see for instance Kawasaki et al. 2019). A large body of papers in the marketing-channel literature define intra-brand competition as the competition between retailers of the same product, while inter-brand competition occurs between manufacturers of substitutable products. This literature is primarily concerned with the coordination of marketing channels, using pricing and/or marketing mix variables (see Cai et al. 2019 for a survey).

Unlike the above-cited literature, we do not assume coordination, integration or agreements between firms. Using the emergence of green production practices as a motivating example, the objective of this paper is to analyze various sources of competition in a differentiated oligopoly. For instance, the introduction of green products in an oligopolistic market corresponds to introducing inter-brand competition in a context where intra-brand competition is present. On the other hand, moving from a differentiated duopoly to a differentiated oligopoly corresponds to introducing intra-brand competition in a context with inter-brand competition.

We extend the duopoly model of Singh and Vives (1984) by assuming that two varieties of a product are supplied by two groups of homogeneous firms that compete in quantity. This is different than having *N* firms producing one variety each, as in Vives (1985), Häckner (2000) or Amir and Jin (2001), since it accounts for both inter-brand competition and intra-brand competition. Furthermore, in our model, each firm pays a fixed cost related to the product variety supplied, which is a *sine qua non* condition to access different markets. Finally, for a given industry size, we allow firms to revise their decision about which product variety they will supply. This decision is based on the different groups’ relative economic performance.

We first analyze the differentiated oligopoly model for the Cournot solution corresponding to a given industry size and composition. We then investigate the impact of changes in three different sources of competition, that is, in the degree of product substitutability, in the total number of firms, and in the industry configuration, specifically examining the consequences of such changes on equilibrium quantities and social welfare. We also characterize the equilibrium industry configuration when firms can change their decision about which product variety to supply. Finally, to derive further insights, we perform some numerical simulations in which, rather than adopting simplifying assumptions on the parameter values, we make conjectures about their relative values, based on what we would see in an industry providing a certified “green” product and a conventional “brown” one.

The first part of our analysis is related to what is done in Saggi and Vettas (2002) and Dou and Ye (2017). Both papers use a special case of the differentiated duopoly model where the two markets for the substitutable varieties are identical. In Dou and Ye (2017), the cost structure of the firms is absent, so that the two groups of firms only differ in terms of size, and results are expressed in terms of market composition only. In Saggi and Vettas (2002), the costs (fixed and variable) and group sizes result from upstream decisions.

In our model we adopt an asymmetric context that encompasses both the Dou and Ye (2017) and Saggi and Vettas (2002) models: firms sustain different production costs, and markets exhibit different features, so that all the model parameters are asymmetric. This allows us to express our results not only in terms of market composition, but also of market conditions and firms’ characteristics. This broad asymmetric context is encapsulated in one parameter called “weighted relative efficiency” (WRE), expressed as the two types of firms showing different WRE levels (e.g. small and large). Symmetric results can be retrieved from ours as a very special case, when firms in both groups have the same WRE, which can still be replicated in our model by firms and markets characterized by different parameter values. This is because, in our differentiated oligopoly, it is the relationship among the parameters that matters rather than their individual values.

In terms of findings, one of our main contributions regards the impact of product substitutability on the individual equilibrium quantities. We find that a stronger horizontal product competition can have a positive effect on a firm’s output in the presence of intra-brand competition, which is not possible in a simple differentiated duopoly. This beneficial effect on a firm’s output is related to the interaction between the degree of product substitutability and the (negative) variation of the other product-type quantity. We identify the market conditions, firms’ characteristics, and industry composition that result in a positive impact of product substitutability on the individual equilibrium quantities.

A second main result comes from the analysis of the social welfare impact of changes in the market composition due to either firm entry or to firms switching from one group to the other. We find that positive impacts are driven by two facets: an increase in the overall industry efficiency, and/or an increase in intra-brand competition.

A third main result concerns the impact of intra-brand competition on the equilibrium supply of individual firms when the size of the industry is fixed, that is, when firms switch from one group to the other. An intensification of intra-brand competition in the smaller group always has a negative effect on the individual quantity produced in this group, no matter its WRE. However, an intensification of intra-brand competition in the larger group can have positive consequences on the individual quantities produced by its members, due to the interplay between the degree of product substitutability and the (positive) impact of inter-brand competition.

When we turn to the equilibrium analysis of the industry composition for a fixed overall industry size, we find that all configurations are possible (single-variety market of any type or market with two varieties), according to the values of the model parameters. Comparing the equilibrium market composition to the social optimum configuration, we obtain that social welfare is always maximized when the majority of firms pertains to the group with the highest WRE, while the equilibrium industry composition rather depends on the relative importance of fixed (e.g. certification) costs.

The paper is organized as follows. Section 2 presents the differentiated oligopoly model with two varieties produced by *N* firms. Section 3 analyzes the impact of product substitutability on the equilibrium solution of the game. Sections 4 and 5 investigate the consequences of intra-brand competition, resulting from changes in the size or composition of the industry, on firms’ equilibrium output and on social welfare, while Sect. 6 characterizes the equilibrium market composition arising when firms can switch from one group to the other. Finally, Sect. 7 presents some numerical illustrations and Sect. 8 concludes the paper.

## A general oligopoly with intra- and inter-brand competition

Consider an industry populated by *N* firms. Producers are divided into two groups of similar types, and members of the same group use the same technology to produce a homogeneous product (e.g. with “high” and “low” ecological footprint). Let $k\in \left\{ H,L\right\}$ index the product type and $n_{k}$ denote the number of producers within group *k*, with $n_{H}+n_{L}=N$. Accordingly, assuming a linear cost function, the total production cost of a quantity $q_{ki}$ of product $k\in \left\{ H,L\right\}$ by producer $i\in \left\{ 1,...,n_{k}\right\}$ is given by

$$\begin{aligned} C_{ki}=f_{k}+m_{k}q_{ki} \end{aligned}$$

where $m_{k}\ge 0$ and $f_{k}\ge 0$ are, respectively, the marginal and fixed production costs.

Since goods produced by firms of a given type are homogeneous, consumers are offered two product varieties. Following Singh and Vives (1984), we assume that the representative consumer has a taste for variety, and that her quadratic utility function is strictly concave and described by

$$\begin{aligned} U\left( Q_{H},Q_{L}\right) =A_{H}Q_{H}+A_{L}Q_{L}-\frac{1}{2}\left( F_{H}Q_{H}^{2}+2SQ_{H}Q_{L}+F_{L}Q_{L}^{2}\right) \text {,} \end{aligned}$$

where $Q_{H}$ and $Q_{L}$ are the total production of the firms of type *H* and *L*, respectively, and where $F_{k}>0$, $A_{k}>0$ and $S\ge 0$ for $k\in \left\{ H,L\right\}$. In the same way as in Häckner (2000), the parameters $A_{k}$ can be interpreted as the quality (vertical) differentiation between product varieties. The parameter $S\ge 0$ is the symmetric degree of substitutability between any pair of varieties. When $S=0$, products *H* and *L* are completely independent, and each group of producers of a given type becomes an independent oligopoly selling a homogeneous product (pure intra-brand competition). The strict concavity of the representative consumer’s utility function assumption implies that

1 $$\begin{aligned} S^{2}<F_{H}F_{L}\text {.} \end{aligned}$$

In addition, we assume that the maximum utility of the consumer is achieved in the positive quadrant, which corresponds to

2 $$\begin{aligned} S<\min \left\{ F_{H}\frac{A_{L}}{A_{H}},F_{L}\frac{A_{H}}{A_{L}}\right\} \text {.} \end{aligned}$$

The inverse demand functions faced by producers of each type are obtained by maximizing the consumers’ surplus

$$\begin{aligned} \max \left\{ CS=U\left( Q_{H},Q_{L}\right) -\left( P_{H}Q_{H}+P_{L}Q_{L}\right) \right\} , \end{aligned}$$

yielding, for $j,k\in \left\{ H,L\right\}$ and $j\ne k$

$$\begin{aligned} P_{k}=A_{k}-F_{k}Q_{k}-SQ_{j}\text {.} \end{aligned}$$

We denote by $E_{k}$, with $k\in \left\{ H,L\right\}$, the quantity $A_{k}-m_{k},$ which is assumed to be strictly positive. The parameter $E_{k}$ depends on quality and cost parameters and can be interpreted as an indicator of efficiency. For example, if $E_{k}>E_{j}$, then type-*k* firms are more efficient than type-*j* firms; this greater efficiency can result from a better quality and/or from a production cost advantage. Producers compete in quantities, both within each group, by selling a homogeneous product (intra-brand competition), and with producers of the other group, by offering a different variety (inter-brand competition).

The optimization problem of a single representative producer *i* of group *k* with $k\in \left\{ H,L\right\}$ is given by

$$\begin{aligned} \max _{q_{ki}\ge 0}\left\{ \pi _{ki}\equiv P_{k}q_{ki}-C_{ki}\right\} \text {. } \end{aligned}$$

Since producers in the same group have identical parameters, we can derive, from the first-order conditions, the reaction functions of each type of producer as

$$\begin{aligned} q_{k}=\frac{E_{k}}{F_{k}\left( n_{k}+1\right) }-\frac{Sn_{j}}{F_{k}\left( n_{k}+1\right) }q_{j}\text { with }j,k\in \left\{ H,L\right\} \text {, }j\ne k \text {.} \end{aligned}$$

The equilibrium output of the oligopoly game is then given by

3 $$\begin{aligned} q_{H}= \frac{F_{L}E_{H}\left( n_{L}+1\right) -SE_{L}n_{L}}{\Omega } \end{aligned}$$

4 $$\begin{aligned} q_{L}= \frac{F_{H}E_{L}\left( n_{H}+1\right) -SE_{H}n_{H}}{\Omega } \end{aligned}$$

5 $$\begin{aligned} \Omega&= F_{H}F_{L}\left( N+1\right) +n_{H}n_{L}\left( F_{H}F_{L}-S^{2}\right) \text {.} \end{aligned}$$

The corresponding equilibrium price for a producer of type $k\in \left\{ H,L\right\}$ is given by

$$\begin{aligned} P_{k}=F_{k}q_{k}+m_{k} \end{aligned}$$

and the equilibrium profit is

$$\begin{aligned} \pi _{k}=F_{k}q_{k}^{2}-f_{k}\text {.} \end{aligned}$$

Finally, the producers’ surplus, consumers’ surplus and total welfare at equilibrium depend on the industry size and composition and are respectively

$$\begin{aligned} PS&= F_{H}\frac{Q_{H}^{2}}{n_{H}}+F_{L}\frac{Q_{L}^{2}}{n_{L}} \\ CS&= \frac{1}{2}\left( F_{H}Q_{H}^{2}+2SQ_{H}Q_{L}+F_{L}Q_{L}^{2}\right) \\ W &= \frac{1}{2}\left( F_{H}Q_{H}^{2}\frac{n_{H}+2}{n_{H}} +2SQ_{H}Q_{L} +F_{L}Q_{L}^{2}\frac{n_{L}+2}{n_{L}}\right) . \end{aligned}$$

For $j,k\in \left\{ H,L\right\}$, $j\ne k$, define the ratio

$$\begin{aligned} \gamma _{k}\equiv F_{j}\frac{E_{k}}{E_{j}}=F_{j}\frac{A_{k}-m_{k}}{ A_{j}-m_{j}}. \end{aligned}$$

The ratio $\gamma _{k}$ is a weighted relative efficiency (WRE) parameter, where the weight is given by the other variety’s price sensitivity to supply.

The WRE parameters summarize the impact of six parameters that affect firms profits in both groups, that is, for $k\in \left\{ H,L\right\}$, parameters $A_{k}$ and $F_{k}$, which characterize consumer’s demand for product *k*, and parameter $m_{k}$, which is its variable cost of production. Thus, the WRE of firms in group *k* is increasing with their efficiency $E_{k}=A_{k}-m_{k}$, decreasing with the efficiency $E_{j}$ of firms in the rival group *j*, and increasing with the price sensitivity $F_{j}$ of the firms in group *j*. Without loss of generality, we assume in the sequel that $\gamma _{L}\le \gamma _{H}$, so that a type-*L* firm is associated with the smallest WRE and a type-*H* firm is associated with the largest WRE. The ratio $\gamma \equiv \frac{\gamma _{H}}{\gamma _{L}}$ characterizes the “distance” between type-*L* and type-*H* firms in terms of efficiency and price sensitivity. A large $\gamma$ indicates that *H*-type firms are much more efficient than type-*L* firms, given market demand conditions. Finally, to alleviate notation, we normalize the value of $\gamma _{L}$ to 1, so that $\gamma _{H}=\gamma \ge 1.$

The equilibrium output of the oligopoly game is then, for $j,k\in \left\{ L,H\right\} ,$ $j\ne k$,

$$\begin{aligned} q_{k}&= E_{j}\frac{\gamma _{k}\left( n_{j}+1\right) -Sn_{j}}{\Omega } \\ \Omega&= \gamma \left( N+1\right) +n_{j}n_{k}\left( \gamma -S^{2}\right) . \end{aligned}$$

We assume that

6 $$\begin{aligned} S<1\le \gamma , \end{aligned}$$

which ensures that $q_{k}>0$ for $k\in \left\{ H,L\right\}$ and for any possible value of the number of producers in each group, and implies Condition (1). Finally, we assume that $f_{k}<F_{k}q_{k}^{2}$ for $k\in \left\{ H,L\right\}$, which ensures that both types of producers participate in the market at equilibrium.

Note that when both types of firms have the same WRE $\left( \gamma =1\right)$, production and profits in the two groups may still differ, as they depend on the values of $n_{k}$ and $E_{k}$, $k\in \left\{ H,L\right\} .$

## Impact of product substitutability

In this section, we investigate the relationship between the *degree of substitutability* *S* and the individual equilibrium quantities (and, consequently, the individual profits). An increase in *S*, that is, an increase in the degree of the products’ substitutability, can be interpreted as a more intense horizontal (inter-brand) product competition. The following proposition shows that the impact of an increase in *S* on individual quantities depends on market conditions, firms characteristics, and industry composition.

### Proposition 1

(i): *If* $\gamma >\frac{n_{H}n_{L}}{\left( n_{H}-1\right) \left( n_{L}+1\right) }$, *then* $\frac{\partial q_{L}}{\partial S}<0$, *and there exists an admissible* $S_{H}\in \left( 0,1\right)$ *such that*
  $$\begin{aligned} \frac{\partial q_{H}}{\partial S}\le & {} 0\text { for }S\in [0,S_{H}]\\ \frac{\partial q_{H}}{\partial S}> & {} 0\text { for }S\in \left( S_{H},1\right) . \end{aligned}$$
(ii): *If* $\gamma <\frac{n_{L}\left( n_{H}+2\right) }{\left( n_{H}+1\right) \left( n_{L}+1\right) },$ *then* $\frac{\partial q_{H}}{ \partial S}<0$, *and there exists an admissible* $S_{L}\in \left( 0,1\right)$ *such that*
  $$\begin{aligned} \frac{\partial q_{L}}{\partial S}\le & {} 0\text { for }S\in [0,S_{L}]\\ \frac{\partial q_{L}}{\partial S}> & {} 0\text { for }S\in \left( S_{L},1\right) . \end{aligned}$$
(iii): *In all other cases*, $\frac{\partial q_{L}}{\partial S}\le 0$ *and* $\frac{\partial q_{H}}{\partial S}\le 0$.

### Proof

See Appendix 9.1. $$\square$$

**Fig. 1:**
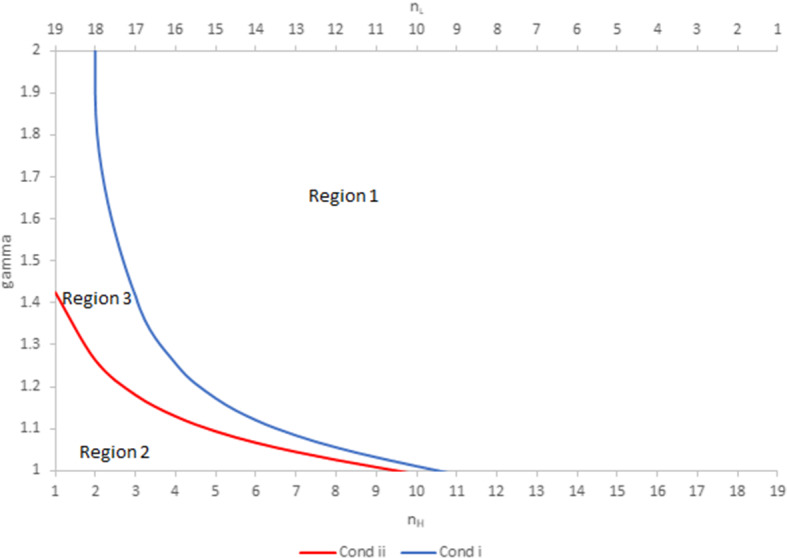
Illustrative example of regions where the substitutability parameter has a positive impact on individual quantities for $N=20$. In Region 1, $\frac{\partial q_{H}}{\partial S}>0$ for sufficiently large *S*. In Region 2, $\frac{\partial q_{L}}{\partial S} >0$ for sufficiently large *S*. In Region 3, both $\frac{\partial q_{H}}{\partial S}$ and $\frac{\partial q_{H}}{\partial S}$ are negative for all *S*

**Fig. 2:**
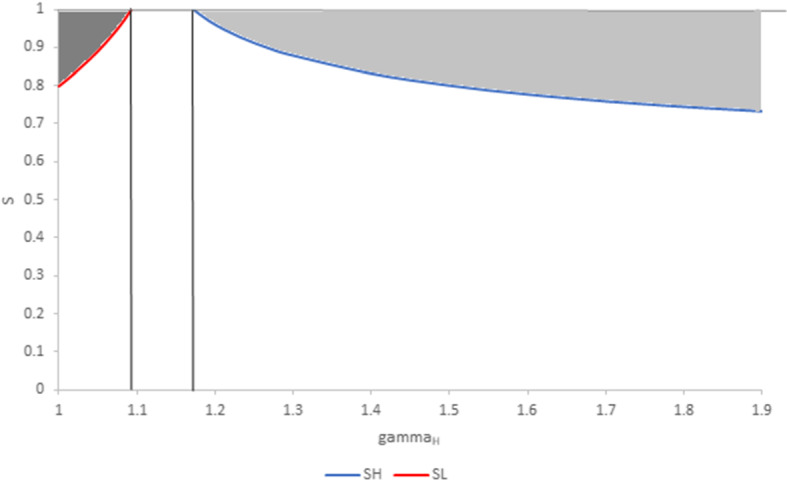
Regions where the substitutability parameter has a positive impact on individual quantities. Parameter values are $n_{L}=15$ and $n_{H}=5$. In the dark grey region, where $\gamma$ is small and $S>$ $S_{L}$, $\frac{dq_{L}}{dS}>0$. In the pale grey region, where $\gamma$ is large and $S>S_{H}$, $\frac{ dq_{H}}{dS}>0$. In all other regions, $\frac{dq_{H}}{dS}<0$ and $\frac{dq_{L} }{dS}<0.$

Proposition 1 states that, under certain conditions, an increase in the degree of product substitutability has a positive impact on the equilibrium quantities produced by individual firms, and consequently on their profit. Results are illustrated in Figs. 1 and 2 for $N=20.$

In Fig. 1, when $\gamma$ is in Region 1, Condition (i) of Proposition 1 is satisfied. In that region, $\frac{\partial q_{L}}{\partial S}<0$ and $\frac{\partial q_{H}}{\partial S}>0$ for sufficiently large *S*. When $\gamma$ is in Region 2, Condition (ii) of Proposition 1 is satisfied. In that region, $\frac{\partial q_{H}}{\partial S}<0$ and $\frac{\partial q_{L}}{\partial S}>0$ for sufficiently large *S*. Finally, when $\gamma$ is in Region 3, both $\frac{ \partial q_{H}}{\partial S}$ and $\frac{\partial q_{H}}{\partial S}$ are negative for all *S*. Region 2 vanishes when $n_{H}\ge n_{L}-1=14$, and Region 3 vanishes when $n_{H}\ge n_{L}+1=16$. The case where both groups have the same WRE is retrieved on the boundary $\gamma =1.$

Figure 2 illustrates the range of values for *S* where substitutability has a positive impact on individual quantities for $n_{L}=15$ and $n_{H}=5$. For small values of $\gamma$ (Region 2), $\frac{ \partial q_{L}}{\partial S}>0$ in the dark grey area. For large values of $\gamma$ (Region 1), $\frac{\partial q_{H}}{\partial S}>0$ in the pale grey area. Elsewhere, both $\frac{\partial q_{L}}{\partial S}$ and $\frac{ \partial q_{H}}{\partial S}$ are negative.

A first insight from Proposition 1 is that the possibility of a stronger horizontal product competition having a positive effect on the individual equilibrium quantities and profit is the specific contribution of intra-brand competition. Indeed, if the model is simplified to a differentiated duopoly (pure inter-brand competition) with $n_{H}=n_{L}=1$, then Conditions *i* and *ii *of Proposition 1 reduce to $\gamma >\frac{1}{0}$ and $\gamma <\frac{3}{4}$ respectively, so that the impact of product substitutability on the equilibrium quantities can never be positive.

A second insight from Proposition 1 relates to the importance of the parameter $\gamma$, which characterizes the WRE advantage of *H*-type firms. When both groups have the same WRE ($\gamma =1$), the impact of an increase in horizontal composition is positive for the largest group. As the value of $\gamma$ increases, the required group size where an increase in *S* can have a positive impact increases for type-*H* firms and decreases for type-*L* firms. Moreover, for a given industry composition satisfying Condition *i*, the lower bound $S_{H}$ is decreasing with $\gamma$, while the lower bound $S_{L}$ is increasing with $\gamma$ for any industry composition.

Finally, note that Conditions *i* and *ii* Proposition 1 are mutually exclusive, so that a positive impact of *S* can not be observed in both groups.

The positive impact of a greater horizontal product competition on the equilibrium quantity of a firm is due to the interplay between the degree of product substitutability and the negative impact it has on the market size of each type of firm. In particular, an increase in *S* reduces the market size of each type of firm and so does each individual quantity ($\frac{\partial q_{k}}{\partial S}<0$ for $k=H,L$). However, if this increase occurs at high enough levels of product substitutability, the reduction of the rival firm’s quantity can outweigh the negative impact that a greater substitutability has on the firm’s market, so that the firm experiences an increase in its equilibrium output.

## Impact of firm entry

We now assess how the equilibrium quantities and corresponding social welfare respond to an intensification of competition. We first focus on the case of an unilateral increase in the number of firms in one group, so that the total number of firms in the industry increases. This scenario can be assimilated to long-term structural changes in the industry size leading to an increase of both inter-brand and intra-brand competition.

It is straightforward to check that a unilateral increase in the number of firms in a given group has a negative impact on the individual output of all the firms in the industry:

$$\begin{aligned} \frac{dq_{k}}{dn_{k}}&= -q_{k}\frac{\left( \gamma -S^{2}\right) n_{j}+\gamma }{\Omega }\le 0 \\ \frac{dq_{j}}{dn_{k}}&= -S\frac{\gamma _{j}E_{k}}{\Omega E_{j}} q_{k}\le 0. \end{aligned}$$

This is due to a general intensification of the competition, specifically intra-brand competition in the expanding group and inter-brand competition with firms in the rival group.

However, the impact of a unilateral increase in the number of firms in a given group on the total output in each market is different: the total equilibrium quantity of the group that experiences a growth in size increases (due to the greater size), but the total equilibrium quantity of the competing group decreases (due to increased inter-brand competition).

$$\begin{aligned} \frac{dQ_{k}}{dn_{k}}&= \gamma \frac{n_{j}+1}{\Omega }q_{k}>0 \\ \frac{dQ_{j}}{dn_{k}}&= -S\frac{\gamma _{j}E_{k}n_{j}}{\Omega E_{j}}q_{k}<0. \end{aligned}$$

It is easy to show that the impact of a unilateral increase in the size of group $k\in \left\{ H,L\right\}$ on the consumers’ surplus is positive for all feasible values of *S* (see Appendix 9.2). This result is expected, since an increase in the number of firms represents a general intensification in competition, which is beneficial to the consumers.^2^

The following proposition gives sufficient conditions for the impact of an increase in the size of the industry to be welfare enhancing.

### Proposition 2

*If* $S=0$ *(independent products), or if* $N=2$ *(differentiated duopoly), the impact of an increase in the size of group* $k\in \left\{ H,L\right\}$ *on the the total welfare is always positive*.

*Otherwise, if*

(i): $\gamma _{k}=1<\gamma$ (*Group* *k* *has the lowest WRE, or, equivalently*, $k=L$ *and* $\gamma _{L}<\gamma _{H}$), *the impact of an increase in* $n_{k}$ *on the welfare is positive for* $S\in [0,1)$ *if*
  $$\begin{aligned} \gamma< & {} \frac{4n_{k}\left( n_{j}+1\right) ^{2}}{n_{j}\left( N+2\right) ^{2}} \\ \text { or }\frac{2n_{k}}{N+2}\le & {} \gamma \le \frac{n_{j}n_{k}}{ n_{j}n_{k}-1}. \end{aligned}$$
(ii): $\gamma =1$ *(both groups have the same WRE), the impact of an increase in* $n_{k}$ *on the welfare is positive for* $S\in [0,1)$ *if*
  $$\begin{aligned} n_{k}<\frac{\left( n_{j}+2\right) ^{2}}{n_{j}}. \end{aligned}$$
(iii): $\gamma _{k}=\gamma >1$ (*Group* *k* *has the highest WRE, or, equivalently*, $k=H$ *and* $\gamma _{L}<\gamma _{H}$), *the impact of an increase in* $n_{k}$ *on the welfare is positive for* $S\in [0,1)$ *if*
  $$\begin{aligned} n_{k}< & {} \frac{\left( n_{j}+2\right) ^{2}}{n_{j}} \\ \text {or }\gamma> & {} \frac{n_{j}\left( N+2\right) ^{2}}{4n_{k}\left( n_{j}+1\right) ^{2}}. \end{aligned}$$

### Proof

See Appendix 9.3. $$\square$$

**Fig. 3:**
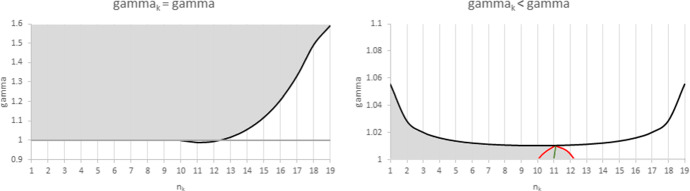
Impact of firm entry for $N=20.$ The left *(resp. right)* panel corresponds to the case where the increasing group has the highest * (resp. lowest)* WRE. The grey areas identify regions where the impact of firm entry on total welfare is positive for any *S*.

The results of Proposition 2 are illustrated in Fig. 3 for $N=20$, where the shaded areas correspond to the regions where an increase in the number of firms in Group *k* is welfare enhancing for any *S*. A positive impact on welfare is driven by two features: an increase in efficiency, and/or an increase in inter-brand competition in the smallest group, where it makes more difference. When the increasing group has the lowest WRE (right panel), the welfare impact can be positive, provided that the size of the increasing group is small and that the difference in efficiency between the two groups is not too large. When the increasing group has the highest WRE (left panel), it is also possible to see a positive impact with an increase in the larger group, provided that its WRE advantage is large enough.

## Impact of changes in the industry composition

We now assume that the total number of firms in the industry is fixed, so that no firm can enter or leave the industry. However, we allow a firm to change the choice of its production technology; this could happen, for instance, for profit considerations. In this case, an increase in the number of firms in one group is compensated for by a decrease in the number of firms in the other group. The group that experiences an increase in size experiences stronger intra-brand competition but reduced inter-brand competition, while the reverse happens in the rival group.

If *N* is assumed constant, then the impact of an increase in the number of firms within group $k\in \left\{ H,L\right\}$ on the total quantity produced by the group is given by

$$\begin{aligned} \frac{dQ_{k}}{dn_{k}}=\gamma _{k}\frac{E_{j}}{\Omega ^{2}}\left( -n_{k}^{2}S^{2}-\gamma _{j}\left( N+1\right) \left( n_{j}-n_{k}\right) S+\gamma \left( n_{j}+1\right) ^{2}\right) , \end{aligned}$$

which is positive for any feasible *S* (see Appendix 9.4). Clearly, when *N* is constant, the impact of an increase in $n_{k}$ is equal to the impact of a decrease in $n_{j}$

$$\begin{aligned} \frac{dQ_{k}}{dn_{k}}=-\frac{dQ_{k}}{dn_{j}}. \end{aligned}$$

Increasing the size of a group decreases the inter-brand competition it experiences: it increases the total quantity produced by this group and decreases the total quantity produced by the other. However, these quantities are divided among a different number of firms, so that individual quantities, and therefore profits, could increase or decrease.

In the following sections, we examine the impact of such a modification in the industry composition on the individual equilibrium quantities and on its social consequences.

### Individual equilibrium quantities

The impact of an increase in the number of firms within group $k\in \left\{ H,L\right\}$ on the individual equilibrium quantity is given by

$$\begin{aligned} \frac{dq_{k}}{dn_{k}}&= -\frac{dq_{k}}{dn_{j}} \\ &= \frac{\partial q_{k}}{\partial n_{k}}-\frac{\partial q_{k}}{\partial n_{j} }\text { with }j,k\in \left\{ H,L\right\} \text { and }j\ne k, \end{aligned}$$

where $\frac{\partial q_{k}}{\partial n_{k}}$ represents the marginal impact of an increase in intra-brand competition and $\frac{\partial q_{k}}{ \partial n_{j}}$ represents the marginal impact of an increase of inter-brand competition.

#### Proposition 3

(i): *If* $$n_{k}\le n_{j}$$,
  $$\begin{aligned} \frac{dq_{k}}{dn_{k}}\le 0\text { for }S\in [0,1). \end{aligned}$$
(ii): *If* $\gamma =1$ *and* $n_{k}>\frac{3N+1}{4}$, *then there exists a* $S_{k}\in (0,1)$ *such that*
  $$\begin{aligned} \frac{dq_{k}}{dn_{k}}>0\text { for }S\in \left( S_{k},1\right) . \end{aligned}$$
(iii): *If* $\gamma _{k}=1<\gamma$ *and* $n_{k}>n_{j}$, *then there exists a* $\overline{S}\in [0,1)$ *such that*
  $$\begin{aligned} \frac{dq_{k}}{dn_{k}}>0\text { for }S\in \left( \overline{S},1\right) . \end{aligned}$$
**(iv)**: *If* $n_{k}>\frac{3N+1}{4}$ *and* $1<\gamma <\frac{3n_{L}^{2}}{ 3n_{L}\left( n_{L}+1\right) -n_{H}+1},$ *then, if* $\gamma$ *is sufficiently small and* $n_{k}$ *is sufficiently large, there exists an admissible range* $\left( S_{1},S_{2}\right) \in [0,1)$ such that
  $$\begin{aligned} \frac{dq_{k}}{dn_{k}}>0\text { for }S_{1}<S<S_{2}. \end{aligned}$$
**(v)**: *In all other cases*, $\frac{dq_{k}}{dn_{k}}<0$ *for all* $S\in [0,1)$ *and* $k\in \left\{ L,H\right\}$.

#### Proof

See Appendix 9.5. $$\square$$

A first result following from Proposition 3 is that, regardless of the market conditions and the firms’ characteristics, a positive impact on the individual equilibrium quantities can only be observed when the group that increases in size is the largest one. This is due to a reduction of the negative impact of inter-brand competition.

It is also important to note that, whenever $\frac{dq_{k}}{dn_{k}}>0$ for a given $k\in \left\{ H,L\right\} ,$ it is also the case that $\frac{dq_{j}}{ dn_{k}}>0$ for $j\ne k$. This is due to the fact that $\frac{dq_{k}}{dn_{k} }>0$ implies $n_{k}>n_{j}$, and therefore $\frac{dq_{j}}{dn_{j}}=-\frac{ dq_{j}}{dn_{k}}<0$. This means that the sign of the marginal impact of a change in the industry composition is the same in both groups.

From Proposition 3 we gather that the impact is positive in both groups when

- the increasing group is much larger than the other, the two groups have the same WRE and products are highly substitutable (Case *ii*);
- the increasing group is the largest, its WRE is the lowest and products are highly substitutable (Case *iii*);
- the increasing group is much larger than the other, its WRE is slightly higher than the other and *S* is inside a range of feasible values (neither high nor low) (Case *iv*).

**Fig. 4:**
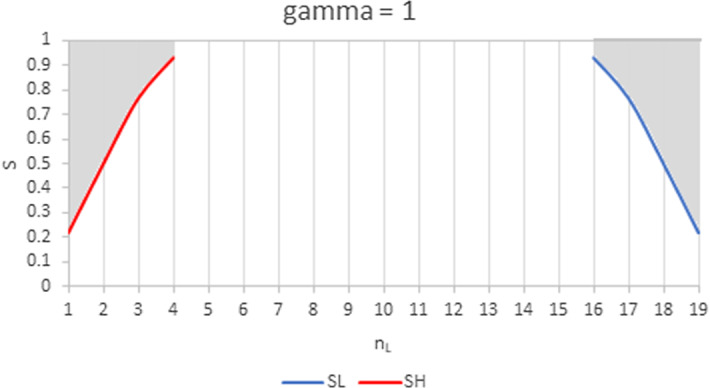
Range of values for *S* and $n_{L}$ where the impact of a change in the industry composition on individual quantities is positive for $N=20$ and $\gamma =1$. In the left shaded area, an increase in $n_{H}$ (i.e. in the larger group) has a positive impact on quantities in both groups. In the right shaded area, an increase in $n_{L}$ has a positive impact on quantities in both groups. For $n_{L}\in \left[ 5,15\right]$, the impact of any shift in the composition of the industry on individual quantities is negative for both groups

**Fig. 5:**
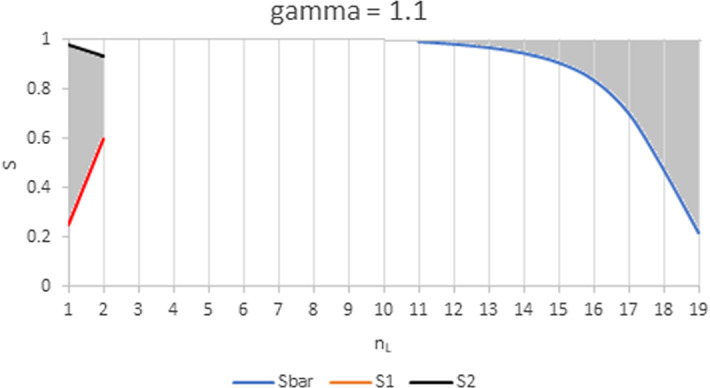
Range of values for *S* and $n_{L}$ where the impact of a change in the industry composition on individual quantities is positive. Parameter values are $N=20$ and $\gamma =1.1$. In the left shaded area, an increase in $n_{H}$, the larger group, has a positive impact on quantities in both groups. In the right shaded area, an increase in $n_{L}$ has a positive impact on quantities in both groups. For $n_{L}\in \left[ 3,10\right]$, the impact of any shift in the composition of the industry on individual quantities is negative for all feasible *S*

**Fig. 6:**
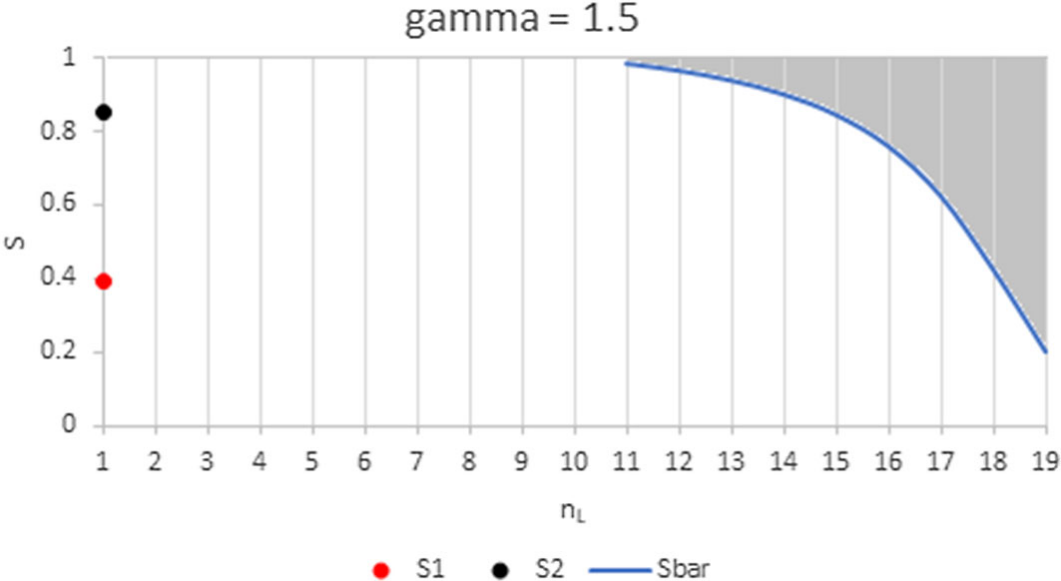
Range of values for *S* and $n_{L}$ where the impact of a change in the industry composition on individual quantities is positive. Parameter values are $N=20$ and $\gamma _{H}=1.5$. In the interval between the two dots, an increase in $n_{H}$, the larger group, has a positive impact on quantities in both groups. In the right shaded area, an increase in $n_{L \text { }}$has a positive impact on quantities in both groups. For $n_{L}\in \left[ 2,10\right]$, the impact of any shift in the composition of the industry on individual quantities is negative for all feasible *S*

Figures 4, 5, 6 illustrate the results of Proposition 3 for $N=20$. The shaded regions are those where a shift from the smaller to the larger group has a positive impact on the individual quantities in both groups. Elsewhere, any change in the industry composition has a negative impact on the individual quantities in both groups.

Figure 4 illustrates Case *i * of Proposition 3: when both groups have the same WRE, a positive impact on quantities can only happen when the increasing group is much larger than the decreasing one ($n_{k}>\frac{3N+1}{4}$). In that case, the impact of the increase in intra-brand competition in the larger group is compensated by the decrease in inter-brand competition. This mechanism increases with *S* and with the discrepancy in group sizes, as can be observed in Fig. 4.

Figures. 5 and 6 show how these areas are affected when the WRE of the two groups are increasingly different. When the largest group is the least efficient ($n_{L}>n_{H}$), the area where an increase in group size has a positive impact on the firms’ output enlarges: less firms are required, and the lower bound $\overline{S}$ shifts downward from $S_{L}$ with increasing $\gamma$. When however the largest group is the most efficient ($n_{H}>\frac{3N+1}{4}>n_{L}$), the area where an increase in group size has a positive impact on the firms’ output shrinks: more firms are required, the lower bound $S_{1}$ shifts upwards from $S_{H}$ and the upper bound $S_{2}$ shifts downward from 1 with increasing $\gamma$. When $\gamma$ is sufficiently high, the left area vanishes and it is no longer possible to obtain a positive impact of an increase in the number of *H*-type firms.

### Social welfare

The following proposition characterizes the impact of a change in the industry composition on consumers’ surplus and total welfare.

#### Proposition 4

*The impact of an increase (resp. decrease) in the number of firms in group* *H* (*resp.* *L*) *on the consumers’ surplus and on the total welfare is positive for any admissible* *S* *when* $n_{H}\le n_{L}$.

*The total welfare admits a unique maximum at* $\widetilde{n}_{H}\in$ $\left[ N/2,N\right]$. *The value of* $\widetilde{n}_{H}$ *is increasing in* $\gamma$.

#### Proof

See Appendix 9.6. $$\square$$

The results of Proposition 4 indicate that an intensification of intra-brand competition has a positive impact on the consumers’ surplus and total welfare when the increasing group is both the smallest and the most efficient. When firms have the same WRE, the welfare maximizing industry composition is at $\widetilde{n}_{H}=\frac{N}{2}$. As $\gamma$ increases, the welfare maximizing composition contains increasingly more *H*-type firms. For very high values of $\gamma$, it may be the case that total welfare is maximized when the industry contains only *H*-type firms.

## Market equilibrium

In this section, we investigate the equilibrium composition of the industry when the total number of firms is fixed, assuming that producers switch from making one variety to the other because of the relative profits of both industries.

Note that when the fixed cost does not differ across producer types, the highest profit in the Cournot competition is achieved by the players with the highest $F_{k}q_{k}^{2}$. This is no longer the case when the production of different varieties generates different fixed production costs; then, comparing the profits of the groups of players is a more complex problem.

The equilibrium quantities and, therefore, the profit of both kinds of producers depend on the composition of the industry, which, for a fixed *N*, can be characterized by $n\equiv n_{H}$.

We define the continuous extensions $\pi _{k}:\left[ 0,N\right] \rightarrow {\mathbb {R}} ,$ $k\in \left\{ H,L\right\}$ of the equilibrium profit of both kinds of producers as a function of *n*. An equilibrium market composition is a number $n^{*}$ such that

7 $$\begin{aligned} \pi _{H}(n^{*})=\pi _{L}(n^{*})\text {.} \end{aligned}$$

The equilibrium condition (7) can be rewritten as

8 $$\begin{aligned} L(n^{*})=R(n^{*}) \end{aligned}$$

where

9 $$\begin{aligned} L(n) &= \left( \gamma +\left( N-n\right) \left( \gamma -S\right) \right) ^{2}-\gamma \left( 1+n\left( 1-S\right) \right) ^{2} \end{aligned}$$

10 $$\begin{aligned} R(n)&= \Omega ^{2}\frac{f_{H}-f_{L}}{E_{L}E_{H}} \nonumber \\ &= \delta \left( \gamma \left( N+1\right) +n\left( \gamma -S^{2}\right) \left( N-n\right) \right) ^{2}. \end{aligned}$$

The parameter $\delta \equiv \frac{f_{H}-f_{L}}{E_{L}E_{H}}$ measures the fixed-cost difference between the two types of technologies, normalized with respect to efficiency values.

We analytically derive conditions on the parameter $\delta$ under which different compositions of the industry arise in equilibrium, as stated in the following proposition.

### Proposition 5

*Define*

$$\begin{aligned} \lambda _{1}\equiv & {} \frac{\gamma -\left( N\left( 1-S\right) +1\right) ^{2} }{\gamma \left( N+1\right) ^{2}} \\ &= \frac{L(N)}{\gamma \left( N+1\right) ^{2}} \\ \lambda _{2}\equiv & {} \frac{-\gamma +\left( N\left( \gamma -S\right) +\gamma \right) ^{2}}{\gamma ^{2}\left( N+1\right) ^{2}} \\ &= \frac{L(0)}{\gamma \left( N+1\right) ^{2}}\text {.} \end{aligned}$$

*At equilibrium*,

(i): *if* $0<\lambda _{2}\le \delta$, *then the industry consists only of type*-*L* *firms*;
(ii): *if* $\lambda _{1}<\delta <\lambda _{2}$, *then the two types of firms coexist in the industry*;
(iii): *otherwise*, $\delta \le \lambda _{1}$ *and the industry consists only of type*-*H* *firms*.

### Proof

See Appendix 9.7. $$\square$$

**Fig. 7:**
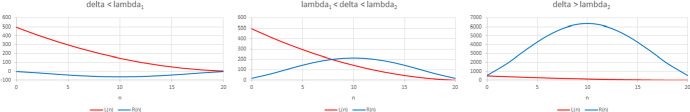
Illustration of the three cases in Proposition 5. Parameter values are $N=20$, $S=0.98$, $\gamma =2.$

Note that the equilibrium solution when the industry is populated by a single type of firm is readily obtained by setting $n_{L}=0$ in (3) or $n_{H}=0$ in (4). Using $R\left( 0\right) =R(N)=\delta \gamma ^{2}\left( N+1\right) ^{2}$, Condition (i) of Proposition 5 implies that $0<L_{0}\le R_{0}$, Condition (ii) implies that $L(N)<R(N)$ and $R(0)<L(0)$, while Condition (iii) implies that $R(N)\le L(N).$ As indicated in the proof of Proposition 5, the monotonicity and convexity properties of functions *L* and *R* allow to obtain the location of their intersection point according to the position of $\delta$, as illustrated in Fig. 7.

**Fig. 8:**
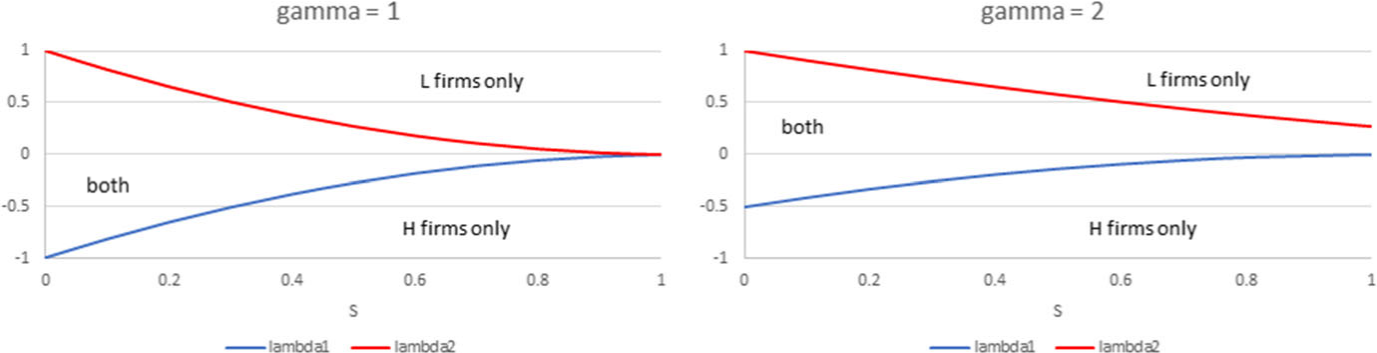
This graph shows the behaviour of $\lambda _{1}$ and $\lambda _{2}$ as a function of *S* and $\gamma$ for $N=20.$

Accordingly, when $\delta \ge \lambda _{2}$, that is, when the fixed cost for *H*-type firms is relatively high, the profit of *L*-type firms is higher than that of *H*-type firms for all possible industry configuration. As a consequence, it is profitable for firms to adopt the *L* technology, and at equilibrium the industry is populated by *L* firms only. The reverse is true when $\delta \le \lambda _{1}$, that is, when the fixed cost for *H* -type firms is relatively low. When $\lambda _{1}<\delta <\lambda _{2}$, then there exists an equilibrium configuration $n^{*}$ where both types of firms coexist and have equal profits. Figure 8 shows how the bounds $\lambda _{1}$ and $\lambda _{2}$ vary with *S* and $\gamma$.

To conclude, it may be interesting to identify policy measures that could lead to a change in the industry composition; in particular, we can identify from the results of Proposition 5 how changes in some parameter values could allow the entry of a new technology, or a change in the proportion of type-*L* and type-*H* firms (see Appendix 9.8). Appropriate policies could be used, for instance, to bring the equilibrium composition closer to the social welfare optimizing composition, as indicated in Proposition 4.

- **P1** **Starting from an industry with only type-***L*** firms**, type-*H* firms appear with increases in $\lambda _{2}$ and decreases in $\delta$, that is :
  1. Decreases in *S* (less substitutable products);
  2. Increases in $\frac{F_{L}}{F_{H}}$ (higher (*resp. lower*) price sensitivity for *L* (*resp. H*) products;
  3. Smaller (*resp. larger*) fixed cost for producers of type *H* ( *resp. of type L*).
- **P2** **Starting from an industry with only type-***H*** firms**, type-*L* firms appear with decreases in $\lambda _{1}$ and increases in $\delta$, that is:
  1. Decreases in *S* (less substitutable products);
  2. Decreases in $\frac{F_{L}}{F_{H}}$ (higher (*resp. lower*) price sensitivity for *H* (*resp. L*) products).
  3. Larger (*resp. smaller*) fixed cost producers of type *H* (* resp. of type L*).
- **P3** **Starting from a mixed industry**, more type-*H* firms appear with decreases in $f_{H}$ or increases in $f_{L}.$

## Application: green and brown products

In this section, we investigate whether or not the results found for the general oligopoly with two varieties can be further refined. Instead of adopting simplifying assumptions on the parameters, we make conjectures about their relative values, and our assumptions are inspired by what we would expect in an industry with a certified “green” product and a conventional “brown” one. Examples of such green products are those accredited by Fairtrade, the Rainforest Alliance, the Forest Stewardship Council (FSC) and the Marine Stewardship Council (MSC).

The adoption of a production practice adequate for green certification is captured by the marginal cost $m_{G}$ and the fixed cost $f_{G}.$ We assume that

$$\begin{aligned} m_{G}>m_{B}\text {,} \end{aligned}$$

that is, that the technology implemented to produce a certified green product is more expensive than the one adopted to make a conventional one. Moreover, the certification process generates a fixed cost that adds up to any other fixed cost borne by a conventional brown producer; so we assume that

$$\begin{aligned} f_{G}>f_{B}\text {.} \end{aligned}$$

On the demand side, we make no prior assumptions on the relative sensitivity of consumers to the price of each product variety, that is,

$$\begin{aligned} F_{G}<=>F_{B}\text {.} \end{aligned}$$

However, we assume that consumers are willing to pay a premium price for a labelled green product. We model this *green premium* by assuming that when a quantity $Q_{G}+Q_{B}$ is produced, the price of the green variety product is higher, for any feasible $Q_{G}$ and $Q_{B}$:

$$\begin{aligned} A_{G}-F_{G}Q_{G}-SQ_{B}>A_{B}-F_{B}Q_{B}-SQ_{G}\text {,} \end{aligned}$$

which translates into a higher choke price, that is,

$$\begin{aligned} A_{G}>A_{B}\text {,} \end{aligned}$$

with the additional condition

$$\begin{aligned} A_{G}F_{B}>A_{B}F_{G}\text {,} \end{aligned}$$

which is always satisfied if $F_{B}\ge F_{G}$. The green premium is then defined as the difference $A_{G}-A_{B}$.

Given these assumptions on the parameters, the stability condition (8) and the comparative statics results remain valid, and we can find numerical examples satisfying all three possibilities listed in Proposition 5 for the steady-state industry composition.

We now provide some numerical illustrations that represent various industry compositions. For comparison purposes, we normalize the values of parameters $A_{B},$ $F_{B}$ and $f_{B}$ in all numerical experiments to $A_{B}=200$, $F_{B}=1$ and $f_{B}=0$, so that $\gamma _{L}$ is no longer necessarily equal to 1.

**Fig. 9:**
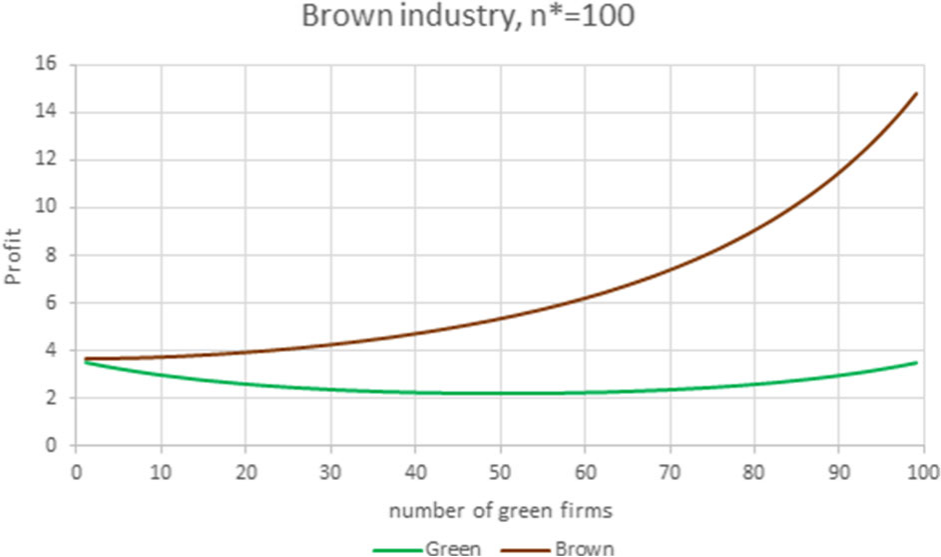
Brown industry. Parameter values are $N=100$, $S=0.99483$, $F_{G}=1$, $A_G=201, m_G=8$, $m_{B}=6$, $f_{G}=0.08$, yielding $E_B=194$, $E_G=193$, $\gamma _{B}=1.00518> \gamma _{G}=0.99485$ and $\gamma =1. 010\,4.$ The equilibrium prices and quantities are $P_{B}=7.92$, $q_{B}=1.92.$

Figure 9 illustrates the case where the firms’ and the market conditions are adverse to the entry of green firms, so that the equilibrium composition of the industry corresponds to a brown one: the WRE advantage of brown firms, measured by $\gamma =\frac{\gamma _{B}}{\gamma _{G}}$, is 1.0104, products are highly substitutable, the marginal cost of production is 33% higher in the green industry, and the green premium is only 0.5% of the choke price. In this particular instance, the equilibrium composition maximizes the total welfare, that is, $\widetilde{n}_{H}=n^{*}=N$.

From Proposition 5, we know that a decrease in the product substitutability and/or a decrease in the brown firms WRE advantage would help green firms enter the market.

**Fig. 10:**
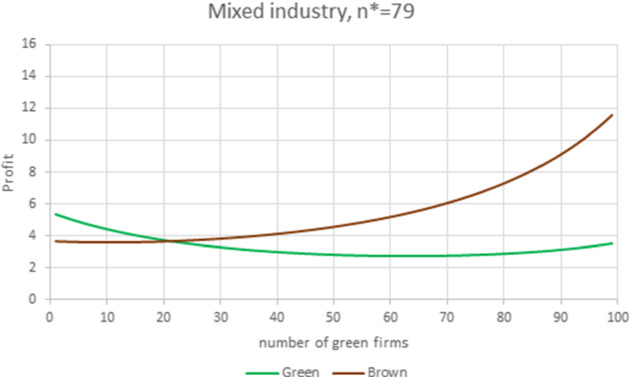
Mixed industry. Parameter values are $N=100$, $S=0.99483$, $F_{G}=1$, $A_G=201.45, m_{G}=8$, $m_{B}=6$, $f_{G}=0.08$, yielding $E_B=194$, $E_G=193.45$, $\gamma _{B}=1.00284> \gamma _{G}=0.99716$ and $\gamma =1.\, 005\,7.$ The equilibrium prices and quantities are $P_{G}=9.94$, $P_{B}=7.92$, $q_{G}=1.94$, $q_{B}=1.92.$

Figure 10 is obtained from Fig. 9 by increasing the green premium, which now accounts for 0.72% of the choke price, and translates into a reduction of the brown firm’s WRE advantage (now $\gamma =1.0057,$ compared to the previous 1.0104). In this example, we have an industry where green firms have spread moderately. At equilibrium, 21 green firms (vs. 79 brown firms) produce 21% of the total quantity, the price of the green product is 26% higher than that of a conventional one, and each green firm produces 1.2% more in terms of quantity than does a brown firm. The additional fixed costs borne by the certified green producers ($f_{G}=0.08$) amount to the 2.2% of the equilibrium profit. In that particular instance, the number of brown firms maximizing the total welfare is $\widetilde{n}_{H}=77$.

**Fig. 11:**
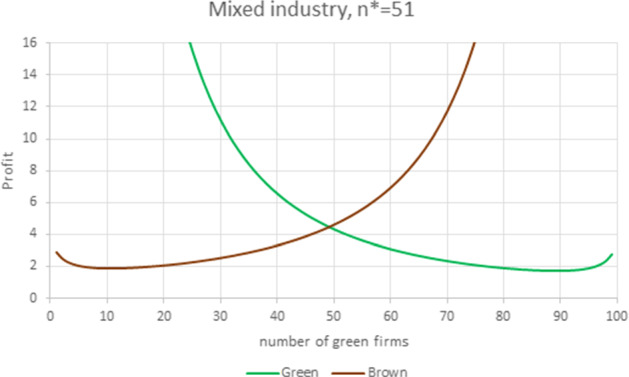
Mixed industry. Parameter values are $N=100$, $S=0.8$, $F_{G}=1$, $A_G=201.45, m_{G}=8$, $m_{B}=6$, $f_{G}=0.08$, yielding $E_B=194$, $E_G=193.45$, $\gamma _{B}=1.00284>\gamma _{G}=0.99716$ and $\gamma =1.\, 005\,7.$ The equilibrium prices and quantities are $P_{G}=10.14$, $P_{B}=8.12$, $q_{G}=2.143$, $q_{B}=2.115.$

Figure 11 illustrates the impact of a lower substitutability between the two product varieties. This could be the result of some (exogenous) investment to make consumers more aware of the difference between the two products, like a more distinctive label or an advertising campaign focused on the green production practices. In this example, all parameter values are the same as in Fig. 10, except for $S=0.8.$ Even if the green WRE is smaller than the brown one, the less aggressive horizontal competition between the two product varieties gives the green products more room in the market. This results in a larger number (49) of green firms as well as a higher profit, selling price and production quantity for both kinds of producers. In this industry, green firms produce 49.3% of the total quantity, the price of the green product is 25% higher than that of the conventional one, and each green firm produces 1.3% more in terms of quantity than does a brown firm. The fixed green certification cost accounts for 1.8% of the equilibrium profit. Note that the number of brown firms that maximizes total welfare in this case is $\widetilde{n} _{H}=51$, so that the equilibrium market composition is optimal.

To have a more substantial presence of green firms in the industry, reducing the fixed cost is not enough; we need the green WRE to be higher than the brown one. This is shown by the next two numerical examples where a high green penetration occurs, regardless of the level of horizontal product competition.

**Fig. 12:**
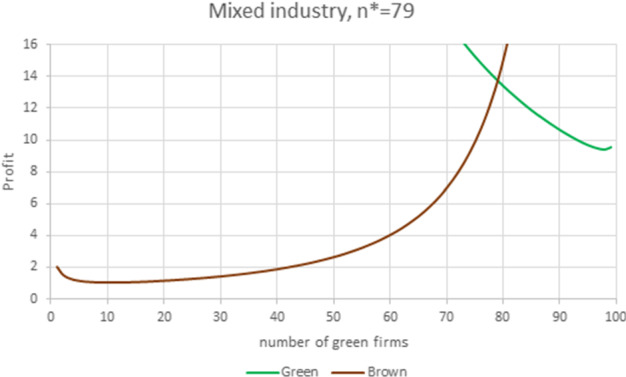
Mixed industry. Parameter values are $N=100$, $S=0.3333$, $F_{G}=1/1.3$, $A_G=297, m_{G}=6$, $m_{B}=5$, $f_{G}=0.5$, yielding $E_B=195$, $E_G=291$, $\gamma _{B}=0.51546< \gamma _{G}=1.49231$ and $\gamma =2.\, 895\,1.$ The equilibrium prices and quantities are $P_{G}=9.31$, $P_{B}=8.71$, $q_{G}=4.307$, $q_{B}=3.709.$

In Fig. 12, green firms have a high WRE advantage, with $\gamma =2.8951$, and the horizontal product competition is weak. This translates into market conditions and a green firm cost structure favorable to a large presence of green firms. Another way to explain the large presence of green firms in the market is to look at the poor performance of the brown firms, which almost nullifies the favorable presence of weak horizontal product competition: even if the two markets are weakly linked, the conditions in the brown market are so adverse that only a few brown firms can survive.

The high green WRE advantage comes from a high green premium, which amounts to 48.5% of the choke price. As a result, the equilibrium price of the green product is 6.9% higher than that of the brown product. At equilibrium, green firms produce 81.4% of the total quantity, each green firm produces 16.1% more than a brown firm and pays a fixed certification cost that amounts to 3.6% of the equilibrium profit. At equilibrium the number of green firms is $n^{*}=79$, while the number of green firms optimizing the total welfare is $\widetilde{n}_{H}=74$.

**Fig. 13:**
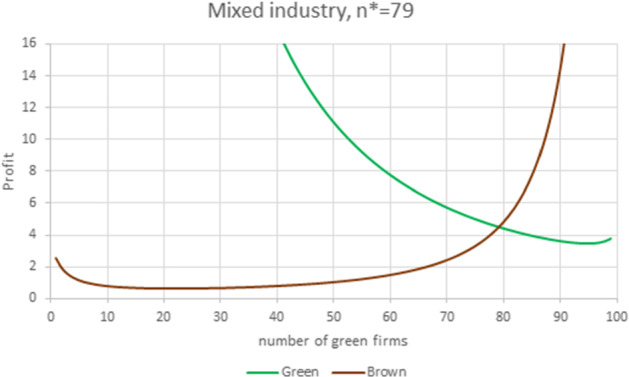
Mixed industry. Parameter values are $N=100$, $S=0.8$, $F_{G}=1/1.1$, $A_G=212.5, m_{G}=6$, $m_{B}=5$, $f_{G}=0.5$, yielding $E_B=195$, $E_G=206.5$, $\gamma _{B}=0.85846< \gamma _{G}=1.05897$ and $\gamma =1.\, 233\,6.$ The equilibrium prices and quantities are $P_{G}=8.14$, $P_{B}=7.10$, $q_{G}=2.354$, $q_{B}=2.101.$

In the last case, illustrated in Fig. 13, green firms have a relatively high WRE advantage ($\gamma =1.2336$) and the horizontal product competition is strong. The large presence of green firms is a consequence of the strong product substitutability: in this case, highly connected markets do not leave much room for inefficient firms. In Fig. 13, 79 green firms produce 80.8% of the total quantity, the price of the green product is 14.6% higher than that of the brown one, with a green premium that accounts for 6.25% of the choke price. Each green firm produces 12% more than does a brown firm, and the certification costs correspond to 11% of the equilibrium profit. The number of green firms maximizing total welfare is $\widetilde{n}_{H}=78$.

From the previous numerical experiments, we can draw some insights related to the equilibrium industry composition. Firstly, a significant presence of green firms is achievable only if their WRE is greater than that of firms producing the rival variety, given our assumptions about their relatively higher fixed costs. Secondly, a specific equilibrium composition can be reached under different combinations of horizontal product competition and WRE advantage. Changes in the fixed certification costs result in a vertical shift of the green firms’ profit function, so that the composition of the industry is more sensitive to changes in the fixed certification costs when the participation of green firms is low.^3^

Finally, our numerical experiments produce results where the equilibrium industry composition is close to the composition maximizing the total welfare. It is worthwhile recalling that the optimal welfare is obtained when the proportion of firms having a WRE advantage is 50% or more, and that this optimal proportion is increasing with the ratio $\gamma$ and independent of the difference in fixed costs borne by the firms. On the other hand, the equilibrium composition depends on the relative values of the fixed costs. Accordingly, it could happen that the equilibrium industry composition be far from the optimal one, specifically when fixed costs are higher in the group having a WRE advantage.

## Conclusion

In this paper, we propose a differentiated oligopoly model with two product varieties made by two groups of firms. This means introducing intra-brand competition in a context where inter-brand competition is present. Markets and firms are fully asymmetric, creating a very general framework. The asymmetry is encapsulated in a parameter called the weighted relative efficiency (WRE), with relative values symbolizing the advantage of a specific group of firms.

After characterizing the equilibrium solution of the Cournot oligopoly, we analyze its response to the degree of product substitutability (horizontal product differentiation). We find that, due to intra-brand competition, a stronger horizontal competition may in some instance have a positive impact on quantities and profits of the industry.

We also study the consequences and welfare impacts of changes in the industry composition. We analyze both unilateral (long-term) changes and changes resulting from industries switching from one group to the other. Such changes are to be understood as the possibility for a firm to adjust its production practice and join the group producing the alternative product variety when the total number of players in the industry is fixed. Assuming that such behavior is driven by profit considerations, we further characterize the equilibrium composition of the market.

Our results depend on the relative WRE of the two types of firms, as well as on the market composition, making them very general and encompassing previous developments found in the literature. Finally, numerical simulations are provided in the context of brown and green production processes, and are used to illustrate theoretical results.

## Appendix

### Proof of Proposition 1

The impact of *S* on the equilibrium quantity of a firm in group *k* is

$$\begin{aligned} \frac{dq_{k}}{dS}&= E_{j}\frac{n_{j}}{\Omega ^{2}} \left( -S^{2}n_{j}n_{k}+2S\gamma _{k}n_{k}\left( n_{j}+1\right) -\gamma \left( n_{k}+1\right) \left( n_{j}+1\right) \right) , \ k,j\in \left\{ L,H\right\} ,\text { }k\ne j \\ &= E_{j}\frac{n_{j}}{\Omega ^{2}} \Phi _{k}(S). \end{aligned}$$

To ease notation, define $\alpha _{k}\equiv \frac{n_{k}+1}{n_{k}}$, $k\in \left\{ H,L\right\}$. $\Phi _{k}(S)$ is a concave parabola with a negative intercept and roots

$$\begin{aligned}&\gamma _{k}\alpha _{j}\pm \sqrt{ \Delta _{k}} \\&\Delta _{k} =\gamma _{k}\alpha _{j}\left( \gamma _{k}\alpha _{j}-\gamma _{j}\alpha _{k}\right) ,\ k,j\in \left\{ L,H\right\} ,k\ne j, \end{aligned}$$

where $\gamma _{k}\alpha _{j}>1$. This means that $\Phi _{k}(S)$ can only be positive if $\Delta _{k}\ge 0$ and if the smallest root $\alpha _{j}\gamma _{k}-\sqrt{\Delta _{k}}$ is an interior feasible value for *S*, that is, $0<\alpha _{j}\gamma _{k}-\sqrt{\Delta _{k}}<1.$

1. When *S* vanishes (products are independent), $\Phi _{k}(S)<0$ for $k\in \left\{ L,H\right\}$.
2. For $S>0$, if $\gamma \alpha _{L}=\alpha _{H}$, then $\Delta _{H}=\Delta _{L}=0$ and $\Phi _{k}(S)<0$ for $k\in \left\{ L,H\right\}$ and $S\in \left( 0,1\right)$.
3. For $S>0$ and $\gamma \alpha _{L}\ne \alpha _{H}$, we will consider two distinct cases.
  - $$\gamma \alpha _{L}>\alpha _{H}$$
    In that case, $\Delta _{H}>0$ and $\Delta _{L}<0$. We then have $\Phi _{L}(S)$ $<0$ and $\Phi _{H}(S)\lessgtr 0$, depending on the value of *S*. The smallest root of $\Phi _{H}(S)$ is
    $$\begin{aligned} S_{H}\equiv \alpha _{L}\gamma -\sqrt{\alpha _{L}\gamma \left( \alpha _{L}\gamma -\alpha _{H}\right) }>0. \end{aligned}$$
    Assume that $\gamma >\frac{n_{L}n_{H}}{\left( n_{L}+1\right) \left( n_{H}-1\right) }$, which satisfies the assumption $\gamma \alpha _{L}>\alpha _{H}:$
    $$\begin{aligned} \gamma \alpha _{L}-\alpha _{H}> & {} \frac{n_{L}n_{H}}{\left( n_{L}+1\right) \left( n_{H}-1\right) }\alpha _{L}-\alpha _{H} \\ &= \frac{1}{n_{H}\left( n_{H}-1\right) }>0. \end{aligned}$$
    Note that this condition is always satisfied for $n_{H}>n_{L}+1.$
    The condition
    $$\begin{aligned} \gamma >\frac{n_{L}n_{H}}{\left( n_{L}+1\right) \left( n_{H}-1\right) } \end{aligned}$$
    is equivalent to
    $$\begin{aligned} \left( \alpha _{L}\gamma -1\right) ^{2}<\alpha _{L}\gamma \left( \alpha _{L}\gamma -\alpha _{H}\right) . \end{aligned}$$
    Taking positive roots on both sides yields
    $$\begin{aligned} \alpha _{L}\gamma -1<\sqrt{\alpha _{L}\gamma \left( \alpha _{L}\gamma -\alpha _{H}\right) }. \end{aligned}$$
    Therefore, the root $S_{H}$ is strictly inside the feasible interval, $0<S_{H}<1$, and $\Phi _{H}>0$ for $S\in$ $\left( S_{H},1\right)$.
    When $\gamma =1,$ condition $\gamma >\frac{n_{L}n_{H}}{\left( n_{L}+1\right) \left( n_{H}-1\right) }$ reduces to $n_{H}>n_{L}+1.$ In that case, $\Phi _{H}(S)>0$ if $n_{H}>n_{L}+1$.
  - $$\gamma \alpha _{L}<\alpha _{H}$$
    In that case, $\Delta _{H}<0$ and $\Delta _{L}>0$. We then have $\Phi _{H}(S)$ $<0$ and $\Phi _{L}(S)\lessgtr 0$, depending on the value of *S*. The smallest root of $\Phi _{L}(S)$ is
    $$\begin{aligned} S_{L}\equiv \alpha _{H}-\sqrt{\alpha _{H}\left( \alpha _{H}-\alpha _{L}\gamma \right) }>0. \end{aligned}$$
    Assume that $\gamma <\frac{n_{L}\left( n_{H}+2\right) }{\left( n_{L}+1\right) \left( n_{H}+1\right) }$, which satisfies the assumption $\gamma \alpha _{L}<\alpha _{H}:$
    $$\begin{aligned} \gamma \alpha _{L}-\alpha _{H}< & {} \frac{n_{L}\left( n_{H}+2\right) }{\left( n_{L}+1\right) \left( n_{H}+1\right) }\alpha _{L}-\alpha _{H} \\ &= -\frac{1}{n_{H}\left( n_{H}+1\right) }<0. \end{aligned}$$
    Note that this condition can only be satisfied for $n_{H}<n_{L}-1$.
    The condition
    $$\begin{aligned} \gamma <\frac{n_{L}\left( n_{H}+2\right) }{\left( n_{L}+1\right) \left( n_{H}+1\right) } \end{aligned}$$
    is equivalent to
    $$\begin{aligned} \left( \alpha _{H}-1\right) ^{2}<\alpha _{H}\left( \alpha _{H}-\alpha _{L}\gamma \right) . \end{aligned}$$
    Taking positive roots on both sides yields
    $$\begin{aligned} \alpha _{H}-1<\sqrt{\alpha _{H}\left( \alpha _{H}-\alpha _{L}\gamma \right) } . \end{aligned}$$
    Therefore, the root $S_{L}$ is strictly inside the feasible interval, $0<S_{L}<1$, and $\Phi _{L}>0$ for $S\in$ $\left( S_{L},1\right)$.
    When $\gamma =1,$ condition $\gamma >\frac{n_{L}n_{H}}{\left( n_{L}+1\right) \left( n_{H}-1\right) }$ reduces to $n_{L}>n_{H}+1.$ In that case, $\Phi _{L}(S)>0$ if $n_{L}>n_{H}+1$.

### Impact of firm entry on consumers’ surplus

The consumers’ surplus is

$$\begin{aligned} CS=\frac{1}{2}\frac{E_{H}}{E_{L}}Q_{H}^{2}+SQ_{H}Q_{L}+\frac{\gamma }{2} \frac{E_{L}}{E_{H}}Q_{L}^{2}. \end{aligned}$$

We then have

$$\begin{aligned} \frac{dCS}{dn_{k}} &= \frac{\partial CS}{\partial Q_{k}}\frac{dQ_{k}}{dn_{k}} +\frac{\partial CS}{\partial Q_{j}}\frac{dQ_{j}}{dn_{k}} \\ &= \left( SQ_{j}+\frac{\gamma _{j}E_{k}}{E_{j}}Q_{k}\right) \gamma \frac{ n_{j}+1}{\Omega }q_{k}-\left( SQ_{k}+\frac{\gamma _{k}E_{j}}{E_{k}} Q_{j}\right) S\frac{\gamma _{j}E_{k}n_{j}}{\Omega E_{j}}q_{k} \\ &= q_{k}\frac{S\gamma E_{j}Q_{j}+\gamma _{j}E_{k}Q_{k}\left( \gamma +n_{j}\left( \gamma -S^{2}\right) \right) }{\Omega E_{j}} , \end{aligned}$$

which is strictly positive for $S<\gamma .$

### Proof of Proposition 2

The impact on total welfare is

$$\begin{aligned} \frac{dW}{dn_{k}} &= \frac{dCS}{dn_{k}}+\frac{dPS}{dn_{k}} \\ &= \frac{\gamma q_{k}}{\Omega E_{j}}\left( \gamma _{j}E_{k}q_{k}\left( n_{j}+1\right) -SE_{j}n_{j}q_{j}\right) \\ &= \frac{E_{k}\gamma q_{k}}{\Omega ^{2}}\Phi (S) \end{aligned}$$

where

$$\begin{aligned} \Phi (S)\equiv n_{j}n_{k}S^{2}-S\gamma _{j}n_{j}\left( N+2\right) +\gamma \left( n_{j}+1\right) ^{2}. \end{aligned}$$

When $$S=0,$$ $$\Phi (0)=\gamma \left( n_{j}+1\right) ^{2}>0$$. When $$N=2,\Phi (S)=4\left( \gamma -S\gamma _{j}\right) +S^{2}>0.$$

Note that $\Phi (S)$ is a convex quadratic function, positive and decreasing at $S=0$, with a minimum at $\frac{1}{2}\gamma _{j}\frac{N+2}{n_{k}}>0.$

The function $\Phi (S)$ is positive on [0, 1) if it has no roots, that is if

11 $$\begin{aligned} \gamma _{j}^{2}n_{j}\left( N+2\right) ^{2}-4n_{k}\gamma \left( n_{j}+1\right) ^{2}<0, \end{aligned}$$

or if it is non-negative and non-increasing at $S=1$, that is if

12 $$\begin{aligned} n_{j}n_{k}-\gamma _{j}n_{j}\left( N+2\right) +\gamma \left( n_{j}+1\right) ^{2}\ge & {} 0 \end{aligned}$$

13 $$\begin{aligned} 2n_{k}-\gamma _{j}\left( N+2\right)\le & {} 0. \end{aligned}$$

We will consider three cases.

(i): $\gamma _{k}<\gamma$ (the increasing group has the smallest WRE). Conditions (11)-(13) become respectively $$\begin{aligned} \gamma< & {} \frac{4n_{k}\left( n_{j}+1\right) ^{2}}{n_{j}\left( N+2\right) ^{2} } \\ \gamma\le & {} \frac{n_{j}n_{k}}{n_{j}n_{k}-1} \\ \gamma\ge & {} \frac{2n_{k}}{N+2} \end{aligned}$$ where the lower bound $\frac{2n_{k}}{N+2}\le 1$ for $n_{k}\le n_{j}+2$ and is larger than the upper bound $\frac{n_{j}n_{k}}{n_{j}n_{k}-1}$ when $n_{k}>n_{j}+2+2/n_{j}.$
(ii): $\gamma =1$ (both groups have the same WRE). Conditions (11)-(13) become respectively $$\begin{aligned} \left( 4n_{j}+n_{j}\left( n_{j}-n_{k}\right) +4\right) \left( n_{j}-n_{k}\right)< & {} 0 \\ n_{j}n_{k}-n_{j}\left( N+2\right) +\left( n_{j}+1\right) ^{2} &= 1>0 \\ n_{k}\le & {} n_{j}+2. \end{aligned}$$

Note that Condition (11) is equivalent to $n_{j}<$ $n_{k}<\frac{ \left( n_{j}+2\right) ^{2}}{n_{j}}$ and that $n_{j}+2<\frac{\left( n_{j}+2\right) ^{2}}{n_{j}}$, so that Conditions (11)-(13) reduce to

$$\begin{aligned} n_{k}<\frac{\left( n_{j}+2\right) ^{2}}{n_{j}}. \end{aligned}$$

(iii): $\gamma _{k}=\gamma >1$ (the increasing group has higher WRE). Conditions (11)-(13) become respectively $$\begin{aligned} \gamma> & {} \frac{n_{j}\left( N+2\right) ^{2}}{4n_{k}\left( n_{j}+1\right) ^{2}} \\ n_{j}n_{k}-n_{j}\left( N+2\right) +\gamma \left( n_{j}+1\right) ^{2}> & {} 1>0\\ n_{k}\le & {} n_{j}+2. \end{aligned}$$ Note that $\frac{n_{j}\left( N+2\right) ^{2}}{4n_{k}\left( n_{j}+1\right) ^{2}}=1+\frac{1}{4}\frac{\left( 4n_{j}+n_{j}^{2}-n_{j}n_{k}+4\right) \left( n_{j}-n_{k}\right) }{n_{k}\left( n_{j}+1\right) ^{2}}$, so that Condition ( 11) is always satisfied if $n_{j}<n_{k}<\frac{\left( n_{j}+2\right) ^{2}}{n_{j}}$.

### Impact of intra-brand competition on total quantities in each group

The total quantity produced by group *k* is given by

$$\begin{aligned} Q_{k}=E_{j}n_{k}\frac{\gamma _{k}\left( N-n_{k}+1\right) -S\left( N-n_{k}\right) }{\gamma \left( N-n_{k}+1\right) \left( n_{k}+1\right) -S^{2}\left( N-n_{k}\right) n_{k}}\text {.} \end{aligned}$$

The impact of an intensification of intra-brand competition on the total quantity produced by the group is then

$$\begin{aligned} \frac{dQ_{k}}{dn_{k}}=\gamma _{k}E_{j}\frac{-S^{2}n_{k}^{2}-S\gamma _{j}\left( n_{j}-n_{k}\right) \left( N+1\right) +\gamma \left( n_{j}+1\right) ^{2}}{\Omega ^{2}}. \end{aligned}$$

We will consider two distinct cases.

(i): $n_{j}\ge n_{k}$ We then have $$\begin{aligned} \frac{dQ_{k}}{dn_{k}} &> \gamma _{k}E_{j}\frac{-\gamma n_{k}^{2}-\gamma \left( n_{j}-n_{k}\right) \left( N+1\right) +\gamma \left( n_{j}+1\right) ^{2}}{\Omega ^{2}} \\ &= \gamma \gamma _{k}E_{j}\frac{N+1}{\Omega ^{2}}>0. \end{aligned}$$
(ii): $n_{j}<n_{k}$ We then have $$\begin{aligned} \frac{dQ_{k}}{dn_{k}}> & {} \gamma _{k}E_{j}\frac{-S^{2}n_{k}^{2}+S^{2}\left( n_{k}-n_{j}\right) \left( N+1\right) +S^{2}\left( n_{j}+1\right) ^{2}}{ \Omega ^{2}} \\ &= S^{2}\gamma _{k}E_{j}\frac{N+1}{\Omega ^{2}}>0. \end{aligned}$$

### Proof of Proposition 3

For $$k,j\in \left\{ H,L\right\}$$ and $$k\ne j,$$

$$\begin{aligned} \frac{dq_{k}}{dn_{k}}=-\frac{dq_{k}}{dn_{j}}=\frac{E_{j}}{\Omega ^{2}}\Phi _{k}(S) \end{aligned}$$

where

$$\begin{aligned} \Phi _{k}(S)\equiv -n_{j}^{2}S^{3}+\gamma _{k}\left( n_{j}-n_{k}+n_{j}^{2}\right) S^{2}+\gamma \left( n_{j}+n_{k}+n_{j}^{2}+1\right) S-\gamma _{k}\gamma \left( n_{j}+1\right) ^{2}. \end{aligned}$$

$\Phi _{k}$ is a third-degree polynomial of *S*, negative and increasing in *S* at $S=0$, which admits two or no positive roots by Descartes’ rule of sign.

1. Assume $n_{k}\le n_{j}$. We then have
  $$\begin{aligned} \gamma _{k}\left( n_{j}-n_{k}+n_{j}^{2}\right) -Sn_{j}^{2}\ge n_{j}^{2}\left( \gamma _{k}-S\right) >0, \end{aligned}$$
  so that, using $S^{2}<\gamma$,
  $$\begin{aligned} \Phi _{k}(S) &= S^{2}\left( \gamma _{k}\left( n_{j}-n_{k}+n_{j}^{2}\right) -Sn_{j}^{2}\right) +S\gamma \left( N+n_{j}^{2}+1\right) -\gamma _{k}\gamma \left( n_{j}+1\right) ^{2} \\< & {} \gamma \left( \gamma _{k}\left( n_{j}-n_{k}+n_{j}^{2}\right) -Sn_{j}^{2}\right) +S\gamma \left( N+n_{j}^{2}+1\right) -\gamma _{k}\gamma \left( n_{j}+1\right) ^{2} \\ &= -\gamma \left( N+1\right) \left( \gamma _{k}-S\right) <0. \end{aligned}$$
  We conclude that $\Phi _{k}(S)<0$ for all feasible *S* when $n_{k}\le n_{j}$ .
2. Assume $n_{k}>n_{j}$. We will distinguish three cases according to the value of $\gamma _{k}$.
  - $$\gamma _{k}=\gamma _{j}=1$$
    In the symmetric case,
    $$\begin{aligned} \Phi _{k}(S)=\left( 1-S\right) \left( S^{2}n_{j}^{2}-S\left( n_{j}-n_{k}\right) -\left( n_{j}+1\right) ^{2}\right) . \end{aligned}$$
    This polynomial has two positive roots, 1 and
    $$\begin{aligned} S_{k}=\frac{1}{2n_{j}^{2}}\left( n_{j}-n_{k}+\sqrt{4n_{j}^{2}\left( n_{j}+1\right) ^{2}+\left( n_{j}-n_{k}\right) ^{2}}\right) >0. \end{aligned}$$
    At $$S=1 ,$$
    $$\begin{aligned} \frac{d\Phi _{k}}{dS}=3n_{j}-n_{k}+1=3N+1-4n_{k}. \end{aligned}$$
    If $3N+1-4n_{k}$ $\ge 0$, then $\Phi _{k}$ is non-decreasing in *S* at $S=1$ , and therefore $S_{k}\ge 1$ and $\Phi _{k}(S)<0$ for all $S\in [0,1) .$
    Otherwise, $n_{k}>\frac{3N+1}{4}$, $S_{k}<1$ and $\Phi _{k}(S)>0$ for $S\in \left( S_{k},1\right) .$
    Therefore $\Phi _{k}(S)>0$ for $S\in \left( S_{k},1\right)$ when $n_{k}> \frac{3N+1}{4}>n_{j}.$
  - $$\gamma _{k}=1<\gamma$$
    At $$S=1,$$
    $$\begin{aligned} \Phi _{k}(1) &= -n_{j}^{2}+\left( n_{j}-n_{k}+n_{j}^{2}\right) +\gamma \left( N+n_{j}^{2}+1\right) -\gamma \left( n_{j}+1\right) ^{2} \\ &= \left( \gamma -1\right) \left( n_{k}-n_{j}\right) >0. \end{aligned}$$
    Therefore $\Phi _{k}>0$ when *S* is large enough.
  - $$\gamma _{k}=\gamma >1$$
    At $$S=1,$$
    $$\begin{aligned} \Phi _{k}(1) &= -n_{j}^{2}+\gamma \left( n_{j}-n_{k}+n_{j}^{2}\right) +\gamma \left( N+n_{j}^{2}+1\right) -\gamma ^{2}\left( n_{j}+1\right) ^{2} \\ &= -\left( \gamma -1\right) \left( \gamma +2\gamma n_{j}+n_{j}^{2}\left( \gamma -1\right) \right) <0 \end{aligned}$$
    and
    $$\begin{aligned} \Phi _{k}^{\prime }(1) &= -3n_{j}^{2}+2\gamma \left( n_{j}-n_{k}+n_{j}^{2}\right) +\gamma \left( N+n_{j}^{2}+1\right) \\ &= \gamma \left( 3n_{j}\left( n_{j}+1\right) -n_{k}+1\right) -3n_{j}^{2}. \end{aligned}$$
    If $\gamma \ge \frac{3n_{j}^{2}}{\left( 3n_{j}\left( n_{j}+1\right) -n_{k}+1\right) }$, $\Phi _{H}^{\prime }(1)\ge 0$ and therefore $\Phi _{H}(S)<0$ for all $S\in [0,1)$.
    Otherwise $1<\gamma <\frac{3n_{j}^{2}}{\left( 3n_{j}\left( n_{j}+1\right) -n_{k}+1\right) },$ which can only happen if $n_{k}>\frac{3N+1}{4}.$
    In that case, $\Phi _{k}(S)$ has either no roots or two positive roots in [0, 1). Since $\Phi _{k}(S)$ has two positive roots at $\gamma =1$ for $n_{k}>\frac{3N+1}{4}$, $\Phi _{k}(S)$ has two positive roots that are smaller than 1 if $\gamma$ is sufficiently small and $n_{k}$ is sufficiently large.

### Welfare impact of a change in industry composition

Clearly, since the total number of firms is constant, the impact of an increase in $n_{k}$ on global quantities (consumers’ surplus and total welfare) is equal to the impact of a decrease in $n_{j}$. Without loss of generality, in this proof we will assume that $k=H$ and $j=L$, so that $\gamma _{k}=\gamma \ge 1$ and $\gamma _{j}=1$.

To alleviate notation, define for $k\in \left\{ H,L\right\}$

$$\begin{aligned} Z_{k} &\equiv \gamma _{k}\left( n_{j}+1\right) -Sn_{j} \\ &= \frac{q_{k}}{E_{j}}\Omega >0. \end{aligned}$$

#### Consumers’ surplus

The impact of an increase in the number of firms in group *k* on the consumers’ surplus is given by

$$\begin{aligned} \frac{dCS}{dn_{k}} &= \frac{\partial CS}{\partial n_{k}}-\frac{\partial CS}{ \partial n_{j}} \\ &= q_{k}\frac{S\gamma E_{j}Q_{j}+E_{k}Q_{k}\left( \gamma +n_{j}\left( \gamma -S^{2}\right) \right) }{\Omega E_{j}} \\&\quad-q_{j}\frac{S\gamma E_{k}Q_{k}+\gamma E_{j}Q_{j}\left( \gamma +n_{k}\left( \gamma -S^{2}\right) \right) }{\Omega E_{k}} \\ &= \frac{E_{j}E_{k}}{\Omega ^{3}}\Psi _{C}\left( \gamma \right) \end{aligned}$$

where

$$\begin{aligned} \Psi _{C}\left( \gamma \right) =\gamma \left( Z_{k}^{2}n_{k}-\gamma Z_{j}^{2}n_{j}\right) +n_{j}n_{k}\left( \gamma -S^{2}\right) \left( Z_{k}^{2}-\gamma Z_{j}^{2}\right) +S\gamma Z_{j}Z_{k}\left( n_{j}-n_{k}\right) . \end{aligned}$$

(i): $n_{j}=n_{k}.$ In that case, $$\begin{aligned} \Psi _{C}\left( \gamma \right) &= n\left( Z_{k}^{2}-\gamma Z_{j}^{2}\right) \left( \gamma +n\left( \gamma -S^{2}\right) \right) \\ &= n\left( \gamma -1\right) \left( \gamma +2n\gamma +n^{2}\left( \gamma -S^{2}\right) \right) \left( \gamma +n\left( \gamma -S^{2}\right) \right) , \end{aligned}$$ so that $\Psi _{C}$ is null at $\gamma =1$ and strictly positive for $\gamma >1.$
(ii): $\gamma =1,$ $n_{j}\ne n_{k}.$ In that case, $$\begin{aligned} \Psi _{C}(1) &= \left( Z_{k}^{2}n_{k}-Z_{j}^{2}n_{j}\right) +n_{j}n_{k}\left( 1-S^{2}\right) \left( Z_{k}^{2}-Z_{j}^{2}\right) +SZ_{j}Z_{k}\left( n_{j}-n_{k}\right) \\ &= \left( n_{j}-n_{k}\right) \left( 1-S\right) \left( n_{j}n_{k}\left( N\left( 1-S\right) +3\right) \left( 1-S^{2}\right) +SN-1\right) \end{aligned}$$ where, using $n_{j}n_{k}=n_{k}\left( N-n_{k}\right) \ge \left( N-1\right)$ and $N\ge 2,$ $$\begin{aligned}&n_{j}n_{k}\left( N\left( 1-S\right) +3\right) \left( 1-S^{2}\right) +SN-1\\&\quad \ge \left( N-1\right) \left( N\left( 1-S\right) +3\right) \left( 1-S^{2}\right) +SN-1 \\&\quad =\left( 1-S^{2}\right) \left( 1-S\right) N^{2}+N\left( 2S\left( 1-S\right) +2-S^{3}\right) +3S^{2}-4 \\&\quad \ge 4\left( 1-S^{2}\right) \left( 1-S\right) +2\left( 2S\left( 1-S\right) +2-S^{3}\right) +3S^{2}-4 \\&\quad =\left( 2-S\right) \left( S+2\left( 1-S^{2}\right) \right) >0. \end{aligned}$$ We conclude that $\Psi _{C}(1)$ is strictly positive if $n_{j}>n_{k}$ and strictly negative if $n_{k}>n_{j}$.
(iii): $\gamma >1,$ $n_{j}>n_{k}.$ We will show that $\Psi _{C}$ is increasing in $\gamma$ when $n_{j}>n_{k}$. Since $\Psi _{C}(1)>0$ for $n_{j}>n_{k}$, this implies that $\Psi _{C}(\gamma )>0$ for $\gamma \ge 1$. $$\begin{aligned} \Psi _{C}\left( \gamma \right)&= \gamma ^{3}n_{k}\left( n_{j}+1\right) ^{3}-\gamma ^{2}n_{j}\left( n_{k}+1\right) ^{3} \\&\quad+\gamma ^{2}S^{2}n_{k}\left( N-n_{j}\left( 3n_{j}+n_{j}^{2}+n_{k}^{2}+3\right) \right) \\&\quad+\gamma ^{2}S\left( n_{j}-n_{k}\right) \left( N-n_{j}n_{k}\left( 2N+3\right) +1\right) \\&\quad+ \gamma S^{3}\left( Sn_{j}n_{k}^{3}+n_{j}n_{k}\left( n_{j}-n_{k}\right) \left( 2N+3\right) \right) \\&\quad+\gamma S^{2}n_{j}\left( -N+n_{k}\left( 3n_{k}+n_{j}^{2}+n_{k}^{2}+3\right) \right) \\&\quad-S^{4}n_{j}^{3}n_{k}. \end{aligned}$$ $\Psi _{C}$ is a third-degree polynomial in $\gamma$. It is increasing in $\gamma$ for $\gamma >1$ if it is increasing and convex at $\gamma =1.$

1. $\Psi _{C}$ is increasing at $\gamma =1.$
  $$\begin{aligned} \Psi _{C}^{\prime }(1)&= S^{4}n_{j}n_{k}^{3}+S^{3}n_{j}n_{k}\left( n_{j}-n_{k}\right) \left( 2N+3\right) \\&\quad-S^{2}\left( 3n_{j}n_{k}\left( 2n_{j}-n_{k}\right) +n_{j}\left( n_{j}+n_{j}^{2}n_{k}+n_{k}^{3}\right) +2n_{k}\left( n_{j}-n_{k}\right) \right) \\&\quad-2S\left( n_{j}-n_{k}\right) \left( -N-1+n_{j}n_{k}\left( 2N+3\right) \right) \\&\quad-2n_{j}+3n_{k}+n_{j}n_{k}\left( 3\left( 3n_{j}-2n_{k}+1\right) +3n_{j}^{2}-2n_{k}^{2}\right) \\ &\equiv \Phi _{C1}(S). \end{aligned}$$
  $\Phi _{C1}(S)$ is positive on $\left[ 0,1\right]$ if it is positive at $S=1$ and decreasing on $\left[ 0,1\right] .$ It is decreasing on $\left[ 0,1 \right]$ if $\Phi _{C1}^{\prime }(0)<0$, $\Phi _{C1}^{\prime }(1)<0$ and $\Phi _{C1}^{\prime \prime \prime }(S)\ge 0.$
  - $$\Phi _{C1}(1)>0$$
    $$\begin{aligned} \Phi _{C1}(1)=n_{k}+n_{j}^{2}+n_{j}n_{k} >0. \end{aligned}$$
  - $$\Phi _{C1}^{\prime }(0)<0$$
    $$\begin{aligned} \Phi _{C1}^{\prime }(0) &= -2\left( n_{j}-n_{k}\right) \left( -N-1+n_{j}n_{k}\left( 2N+3\right) \right) \\< & {} -2\left( n_{j}-n_{k}\right) \left( -N-1+\left( N-1\right) \left( 2N+3\right) \right) \\ &= -4\left( n_{j}-n_{k}\right) \left( N^{2}-2\right) <0. \end{aligned}$$
  - $$\Phi _{C1}^{\prime }(1)<0$$
    $$\begin{aligned} \Phi _{C1}^{\prime }(1) &= 4n_{j}n_{k}^{3}+3n_{j}n_{k}\left( n_{j}-n_{k}\right) \left( 2N+3\right) \\&\quad-2\left( 3n_{j}n_{k}\left( 2n_{j}-n_{k}\right) +n_{j}\left( n_{j}+n_{j}^{2}n_{k}+n_{k}^{3}\right) +2n_{k}\left( n_{j}-n_{k}\right) \right) \\&\quad-2\left( n_{j}-n_{k}\right) \left( -N+n_{j}n_{k}\left( 2N+3\right) -1\right) \\ &= 2\left( n_{j}-n_{k}\right) -2n_{k}\left( 2n_{j}-n_{k}\right) -3n_{j}n_{k}\left( 3n_{j}-n_{k}\right) \\ &= < -2\left( n_{k}-1\right) \left( n_{j}-n_{k}\right) -3n_{j}n_{k}\left( 3n_{j}-n_{k}\right) <0. \end{aligned}$$
  - $$\Phi _{C1}^{\prime \prime \prime }(S)\ge 0$$
    $$\begin{aligned} \Phi _{C1}^{\prime \prime \prime }(S)=24Sn_{j}n_{k}^{3}+6n_{j}n_{k}\left( n_{j}-n_{k}\right) \left( 2N+3\right) >0. \end{aligned}$$
    We conclude that $\Psi _{C}$ is increasing at $\gamma =1.$
2. $\Psi _{C}$ is convex. Now, to show that $\Psi _{C}\left( \gamma \right)$ is convex at 1,
  $$\begin{aligned} \Psi _{C}^{\prime \prime }(1)&= 6n_{k}\left( n_{j}+1\right) ^{3}-2n_{j}\left( n_{k}+1\right) ^{3} \\&\quad-2S^{2}n_{k}\left( 2n_{j}-n_{k}+n_{j}n_{k}^{2}+3n_{j}^{2}+n_{j}^{3}\right) \\&\quad+2S\left( n_{j}-n_{k}\right) \left( N+1-n_{j}n_{k}\left( 2N+3\right) \right) \\\equiv & {} \Phi _{C2}(S) . \end{aligned}$$
  $\Phi _{C2}(S)$ is positive on $\left[ 0,1\right]$ if $\Phi _{C2}(0)>0,$ $\Phi _{C2}(1)>0$ and $\Phi _{C2}^{\prime \prime }(S)\le 0.$
  - $$\Phi _{C2}(0)>0$$
    $$\begin{aligned} \Phi _{C2}(0)&= 2\left( 3n_{k}-n_{j}+n_{j}n_{k}\left( 9n_{j}-3n_{k}+3n_{j}^{2}-n_{k}^{2}+6\right) \right) \\> & {} 2\left( 3n_{k}-\left( N-1\right) +\left( N-1\right) \left( 9n_{j}-3n_{k}+3n_{j}^{2}-n_{k}^{2}+6\right) \right) \\ &= 2\left( 3n_{k}+\left( N-1\right) \left( 9n_{j}-3n_{k}+3n_{j}^{2}-n_{k}^{2}+5\right) \right) >0. \end{aligned}$$
  - $$\Phi _{C2}(1)>0$$
    $$\begin{aligned} \Phi _{C2}(1)=2\left( 2n_{k}+3n_{j}^{2}n_{k}+n_{j}^{2}+4n_{j}n_{k}\right) >0. \end{aligned}$$
  - $$\Phi _{C2}^{\prime \prime }(S)\le 0$$
    $$\begin{aligned} \Phi _{C2}^{\prime \prime }(S)=-4n_{k}\left( 2n_{j}-n_{k}+n_{j}n_{k}^{2}+3n_{j}^{2}+n_{j}^{3}\right) <0. \end{aligned}$$

We conclude that the impact on the consumers’ surplus is strictly positive when $n_{j}>n_{k}$ for $\gamma >1$.

(iv): $\gamma >1,$ $n_{j}<n_{k}$ We have shown that $\Psi _{C}(\gamma )$ is strictly positive at $n_{j}=n_{k}$ for $\gamma >1$. Since $\Psi _{C}$ is a third degree polynomial in $\gamma ^{3}$ and its third derivative is positive, $\Psi _{C}(\gamma )>0$ for all $n_{k}$ when $\gamma$ is sufficiently large. For intermediate values of $\gamma$, $\Psi _{C}(\gamma )$ may be negative for $n_{j}<n_{k}$.

#### Total welfare

The impact of an increase in the number of firms in group $k=H$ on the total welfare is

$$\begin{aligned} \frac{dW}{dn_{k}}=\frac{E_{j}E_{k}}{\Omega ^{3}}\gamma \Psi _{W}(\gamma ) \end{aligned}$$

where

$$\begin{aligned} \Psi _{W}(\gamma )=Z_{k}^{2}\left( n_{j}+1\right) -\gamma Z_{j}^{2}\left( n_{k}+1\right) -SZ_{j}Z_{k}\left( n_{j}-n_{k}\right) . \end{aligned}$$

(i): $n_{k}=n_{j}$ In that case, $$\begin{aligned} \Psi _{W}(\gamma ) &= \left( Z_{k}^{2}-\gamma Z_{j}^{2}\right) \left( n+1\right) \\ &= \left( n+1\right) \left( \gamma -1\right) \left( \gamma \left( n+1\right) ^{2}-S^{2}n^{2}\right) \ge 0, \end{aligned}$$ with $\Psi _{W}(\gamma )>0$ for $\gamma >1.$
(ii): $\gamma =1,$ $n_{k}\ne n_{j}$ In that case, $$\begin{aligned} \Psi _{W}(1)=\left( 1-S\right) \left( n_{j}-n_{k}\right) \Phi _{W1}(S) \end{aligned}$$ where $$\begin{aligned} \Phi _{W1}(S)\equiv N\left( N\left( 1-S\right) -2S+3\right) +3-n_{j}n_{k}\left( 1-S^{2}\right) . \end{aligned}$$ Using $n_{j}n_{k}<\frac{N^{2}}{4},$ $$\begin{aligned} \Phi _{W1}(S)> & {} N\left( N\left( 1-S\right) -2S+3\right) +3-\frac{N^{2}}{4} \left( 1-S^{2}\right) \\ &= \frac{1}{4}\left( N\left( 3-S\right) +6\right) \left( N\left( 1-S\right) +2\right) >0. \end{aligned}$$ We conclude that, when $\gamma =1,$the impact on total welfare is strictly positive if $n_{j}>n_{k}$ and strictly negative if $n_{j}<n_{k}$.
(iii): $\gamma >1,$ $n_{j}>n_{k}$ We will show that $\Psi _{W}(\gamma )$ is increasing in $\gamma$ for $n_{j}>n_{k}.$ $$\begin{aligned} \Psi _{W}(\gamma ) &= \gamma ^{2}\left( n_{j}+1\right) ^{3} \\&\quad+\gamma \left( S^{2}n_{k}\left( \left( n_{j}-n_{k}\right) \left( N+1\right) -n_{k}\left( n_{j}+1\right) \right) -\left( n_{k}+1\right) ^{3}\right) \\&\quad-\gamma S\left( n_{j}-n_{k}\right) \left( 5N+2N^{2}-n_{j}n_{k}+3\right) \\&\quad+S^{2}n_{j}\left( \left( N-Sn_{k}+1\right) \left( n_{j}-n_{k}\right) +n_{j}\left( n_{k}+1\right) \right) \end{aligned}$$ $\Psi _{W}(\gamma )$ is a convex quadratic function. It is increasing on $[1,\infty )$ if $\Psi _{W}^{\prime }(1)>0.$ It comes $$\begin{aligned} \Psi _{W}^{\prime }(1) &= 2\left( n_{j}+1\right) ^{3}-\left( n_{k}+1\right) ^{3} \\&\quad+S^{2}n_{k}\left( \left( n_{j}-n_{k}\right) \left( N+1\right) -n_{k}\left( n_{j}+1\right) \right) \\&\quad-S\left( n_{j}-n_{k}\right) \left( 5N+2N^{2}-n_{j}n_{k}+3\right) \\ &\equiv \Phi _{W2}(S). \end{aligned}$$ Note that $\Phi _{W2}(S)$ is a quadratic function of *S*, with $$\begin{aligned} \Phi _{W2}(0)=2\left( n_{j}+1\right) ^{3}-\left( n_{k}+1\right) ^{3}>0 \end{aligned}$$ and $$\begin{aligned} \Phi _{W2}(1)=3n_{j}+n_{j}^{2}+n_{j}n_{k}+1>0, \end{aligned}$$ Therefore $\Phi _{W2}(S)>0$ for all $S\in [0,1)$ if $\Phi _{W2}^{\prime }(S)<0$ on [0, 1). $$\begin{aligned} \Phi _{W2}^{\prime }(S)&= 2Sn_{k}\left( \left( n_{j}-n_{k}\right) \left( N+1\right) -n_{k}\left( n_{j}+1\right) \right) \\&\quad-\left( n_{j}-n_{k}\right) \left( 5N+2N^{2}-n_{j}n_{k}+3\right) . \end{aligned}$$ If $$\begin{aligned} \left( n_{j}-n_{k}\right) \left( N+1\right) -n_{k}\left( n_{j}+1\right) \le 0, \end{aligned}$$ then $\Phi _{W2}^{\prime }(S)<0$. Otherwise, $$\begin{aligned} \Phi _{W2}^{\prime }(S)< & {} 2n_{k}\left( \left( n_{j}-n_{k}\right) \left( N+1\right) -n_{k}\left( n_{j}+1\right) \right) \\&\quad-\left( n_{j}-n_{k}\right) \left( 5N+2N^{2}-n_{j}n_{k}+3\right) \\ &= -n_{j}\left( 2n_{j}+3\right) \left( n_{j}+1\right) +n_{j}n_{k}\left( n_{j}-n_{k}+2\right) +n_{k}\left( n_{k}+3\right) . \end{aligned}$$ Using $n_{j}>\frac{N}{2},$ $n_{j}n_{k}<\frac{N^{2}}{4}$, $n_{j}-n_{k}<N-2$ and $n_{k}<\frac{N}{2},$ we get $$\begin{aligned} \Phi _{W2}^{\prime }(S)< & {} -\frac{1}{4}N\left( N+2\right) \left( N+3\right) \\&\quad+\frac{1}{4}N^{3}+\frac{1}{4}N\left( N+6\right) \\ &= -N^{2}<0. \end{aligned}$$ We conclude that $\Psi _{W}(\gamma )$ is increasing in $\gamma$ for $n_{j}>n_{k}$. Since it is strictly positive at $\gamma =1$, we can conclude that the impact of an increase in $n_{k}$ is welfare enhancing in this case.
(iv): $\gamma >1,$ $n_{j}<n_{k}$. We have shown that $\Psi _{W}(\gamma )$ is strictly positive on $(1,\infty )$ for any admissible *S* when $n_{j}=n_{k}$. Now we show that for a given $\gamma$ and *S*, $\Psi _{W}$ is decreasing in $n_{k}$ on $\left[ \frac{N}{2},N\right] .$ $$\begin{aligned} \frac{d\Psi _{W}}{dn_{k}} &= -3n_{k}^{2}\left( \gamma -S^{2}\right) \left( \gamma -S+1-S\right) \\&\quad+6n_{k}\left( \gamma -S^{2}\right) \left( \gamma -1+N\left( \gamma -S\right) \right) \\&\quad-N^{2}\left( 3\gamma -S\left( S+2\right) \right) \left( \gamma -S\right) \\&\quad+N\left( 2\gamma \left( 5S-3\gamma \right) +S^{2}\left( \gamma -5\right) \right) +3\gamma \left( 2S-\gamma -1\right) . \end{aligned}$$ This is a second degree polynomial in $n_{k}$, maximized at $n_{k}=\left( \frac{\gamma -1+N\left( \gamma -S\right) }{\gamma +1-2S}\right)$, so that $$\begin{aligned} \frac{d\Psi _{W}}{dn_{k}}\le & {} 3\left( \gamma -S^{2}\right) \frac{\left( \gamma -1+N\left( \gamma -S\right) \right) ^{2}}{\gamma +1-2S} \\&\quad-N^{2}\left( 3\gamma -S\left( S+2\right) \right) \left( \gamma -S\right) \\&\quad+N\left( 2\gamma \left( 5S-3\gamma \right) +S^{2}\left( \gamma -5\right) \right) +3\gamma \left( 2S-\gamma -1\right) \\ &= S\left( N\left( 1-S\right) +1\right) \\&\quad-\gamma \left( N\left( 1-S\right) +2-S\right) \frac{N\left( 1-S\right) \left( \gamma -S\right) +\left( N+3\right) \left( \gamma -S+\gamma \left( 1-S\right) \right) }{\gamma +1-2S} \\< & {} S\left( N\left( 1-S\right) +1\right) \\&\quad-\left( N\left( 1-S\right) +2-S\right) \frac{N\left( 1-S\right) \left( \gamma -S\right) +\left( N+3\right) \left( \gamma -S+\gamma \left( 1-S\right) \right) }{\gamma +1-2S} \\ &= -\left( 1-S\right) \left( N\left( 1-S\right) +2\right) \frac{N\left( 1-S\right) \left( \gamma -S\right) +\left( N+3\right) \left( \gamma -S+\gamma \left( 1-S\right) \right) }{\left( \gamma -S\right) +\left( 1-S\right) } \\< & {} 0. \end{aligned}$$

Therefore, $\Psi _{W}$ changes sign at most once on $\left[ \frac{N}{2},N \right]$, which implies that there is a unique value $\widetilde{n}_{H}\in \left[ \frac{N}{2},N\right]$ maximizing the total welfare, and that the impact of an increase in increase in the number of firms in group $k=H$ on the total welfare is positive when $n_{H}<\widetilde{n}_{H}$ and negative when $n_{H}>\widetilde{n}_{H}$.

### Proof of Proposition5

We first show that $\lambda _{2}>0$ and $\lambda _{2}>\lambda _{1}.$

$$\begin{aligned} \lambda _{2} &= \frac{-\gamma +\left( \gamma \left( N+1\right) -NS\right) ^{2}}{\gamma ^{2}\left( N+1\right) ^{2}} \\\ge & {} \frac{-\gamma +\left( \gamma +N\left( 1-S\right) \right) ^{2}}{\gamma ^{2}\left( N+1\right) ^{2}} \\> & {} \frac{\gamma \left( \gamma -1\right) }{\gamma ^{2}\left( N+1\right) ^{2}} \ge 0.\\ \lambda _{2}-\lambda _{1} &= \frac{N}{\gamma ^{2}\left( N+1\right) ^{2}}h(S), \end{aligned}$$

where

$$\begin{aligned} h(S)=S^{2}N\left( \gamma +1\right) -4S\gamma \left( N+1\right) +\gamma \left( \gamma +1\right) \left( N+2\right) \end{aligned}$$

is a quadratic convex function of *S*, minimized at

$$\begin{aligned} S^{*}=2\gamma \frac{N+1}{N\left( \gamma +1\right) }>1. \end{aligned}$$

Therefore, for $$S<1,$$

$$\begin{aligned} h(S)> & {} h(1) \\ &= \left( \gamma -1\right) \left( 2\gamma +N\left( \gamma -1\right) \right) \\\ge & {} 0 \end{aligned}$$

and $$\lambda _{2}>\lambda _{1}$$.

Compute

$$\begin{aligned} R(0) &= R(N)=\delta \gamma ^{2}\left( N+1\right) ^{2} \\ R^{\prime }(n) &= 2\delta \left( N-2n\right) \left( \gamma -S^{2}\right) \left( n\left( N-n\right) \left( \gamma -S^{2}\right) +\gamma \left( N+1\right) \right) . \end{aligned}$$

If $\delta \ne 0,$ according to the sign of $\delta$, the function *R*(*n*) is a strictly positive (*resp. negative*) fourth-degree polynomial, symmetric w.r.t. $\frac{N}{2}$, increasing (*resp. decreasing*) over $[0,\frac{N}{2})$ and decreasing (*resp. increasing*) over $(\frac{N}{2} ,N].$

The function *L*(*n*) is a quadratic function of *n*, with $L(0)>0$. Compute

$$\begin{aligned} L^{\prime }(n) &= -\left( 2\left( N-n\right) \left( \gamma -S\right) ^{2}+2n\gamma \left( 1-S\right) ^{2}+2\gamma \left( \gamma +1-2S\right) \right) <0 \\ L^{\prime \prime }(n) &= 2\left( \gamma -S^{2}\right) \left( \gamma -1\right) \ge 0. \end{aligned}$$

This shows that *L* is a convex, strictly decreasing function of *n*, with $L(0)>L(N)$. An equilibrium market composition $n^{*}$ is defined by the intersection of a strictly decreasing function with the symmetric function *R*(*n*). Compute

$$\begin{aligned} L(N)-R(N)&= \left( \gamma ^{2}-\gamma \left( 1+N\left( 1-S\right) \right) ^{2}\right) -\delta \gamma ^{2}\left( N+1\right) ^{2} \\ &= \gamma ^{2}\left( N+1\right) ^{2}\left( \lambda _{1}-\delta \right) \\ L(0)-R(0)&= \left( \left( \gamma +N\left( \gamma -S\right) \right) ^{2}-\gamma \right) -\delta \gamma ^{2}\left( N+1\right) ^{2} \\ &= \gamma ^{2}\left( N+1\right) ^{2}\left( \lambda _{2}-\delta \right) \end{aligned}$$

We distinguish the following four cases.

**Case 1:** $$0<\lambda _{2}\le \delta$$

In this case, $L(0)-R(0)\le 0$. Since $L(n)<L(0)\le R(0)<R(n)$ for all $n\in (0,N)$, the profit of firms of type *H* is smaller than that of type-*L* firms for any industry composition, so that there are only firms of type *L* in the industry at equilibrium.

**Case 2: **$$\lambda _{1}<\delta <\lambda _{2}$$

In this case, $L(0)>R(0)$ and $L(N)<R(N)$. Since *L* and *R* are continuous functions, there exists at least one $n^{*}\in \left( 0,N\right)$ where $L(n^{*})=R(n^{*})$; since *L* is strictly decreasing, if $\delta =0$ , the intersection point is unique. Otherwise, there are at most two intersection points, and at most one in the region where *R* is increasing. This implies that if $\delta >0$, since $L(N)<R(N)$, it is not possible to have two intersection points in $\left( 0,N\right)$. This is also the case if $\delta <0$, since $L(0)>R(0)$. Consequently, there is a unique equilibrium market composition value $n^{*}$ where both types of firms coexist.

**Case 3: **$$\delta \le \lambda _{1}$$ and $$\delta \le 0$$

In this case, $L(N)\ge R(N)$ and $R(n)<R(N)\le L(N)<L(n)$ for all $n\in \left( 0,N\right) .$ The profit of firms of type *H* is larger than that of type-*L* firms for any industry composition, so that there are only firms of type *H* in the industry at equilibrium.

**Case 4: **$$0<\delta \le \lambda _{1}$$

In this case, $L(N)-R(N)\ge 0$. We will show that $L(n)-R(n)$ is strictly decreasing on $\left[ 0,N\right]$, which implies that $L(n)-R(n)>0$ for all $n\in [0,N)$ and, consequently, there are only firms of type *H* in the industry at equilibrium.

We have

$$\begin{aligned} L^{\prime }(n)-R^{\prime }(n) &= -2\left( \left( N-n\right) \left( \gamma -S\right) ^{2}+n\gamma \left( 1-S\right) ^{2}+\gamma \left( \gamma +1-2S\right) \right) \\&\quad-2\delta \left( N-2n\right) \left( \gamma -S^{2}\right) \left( n\left( N-n\right) \left( \gamma -S^{2}\right) +\gamma \left( N+1\right) \right) . \end{aligned}$$

(i): If $$N-2n$$ $$\ge 0$$, then $$L^{\prime }(n)-R^{\prime }(n)<0.$$
(ii): Assume $N-2n$ $<0$, so that $$\begin{aligned} L^{\prime }(n)-R^{\prime }(n) &= -2\left( \left( N-n\right) \left( \gamma -S\right) ^{2}+n\gamma \left( 1-S\right) ^{2}+\gamma \left( \gamma +1-2S\right) \right) \\&\quad+2\delta \left( 2n-N\right) \left( \gamma -S^{2}\right) \left( n\left( N-n\right) \left( \gamma -S^{2}\right) +\gamma \left( N+1\right) \right) \\\le & {} -2\left( \left( N-n\right) \left( \gamma -S\right) ^{2}+n\gamma \left( 1-S\right) ^{2}+\gamma \left( \gamma +1-2S\right) \right) \\&\quad+2\lambda _{1}\left( 2n-N\right) \left( \gamma -S^{2}\right) \left( n\left( N-n\right) \left( \gamma -S^{2}\right) +\gamma \left( N+1\right) \right) . \end{aligned}$$ Using $$\begin{aligned} \lambda _{1} &= \frac{\gamma -\left( N\left( 1-S\right) +1\right) ^{2}}{ \gamma \left( N+1\right) ^{2}} \\< & {} \frac{\gamma -1}{\gamma \left( N+1\right) ^{2}},\\ \frac{L^{\prime }(n)-R^{\prime }(n)}{2}< & {} -\left( \left( N-n\right) \left( \gamma -S\right) ^{2}+n\gamma \left( 1-S\right) ^{2}+\gamma \left( \gamma +1-2S\right) \right) \\&\quad+\frac{\left( 2n-N\right) \left( \gamma -S^{2}\right) \left( \gamma -1\right) }{\gamma \left( N+1\right) ^{2}}\left( n\left( N-n\right) \left( \gamma -S^{2}\right) +\gamma \left( N+1\right) \right) \\ &= W(n). \end{aligned}$$ Note that *W* is increasing in *n* : $$\begin{aligned} \frac{dW}{dn}= \frac{\left( \gamma -S^{2}\right) \left( \gamma -1\right) }{\gamma \left( N+1\right) ^{2}}\left( 6n\left( \gamma -S^{2}\right) \left( N-n\right) +S^{2}N^{2}+\gamma \left( 4N+3\right) \right) >0, \end{aligned}$$ so that $$\begin{aligned} W(n)< & {} W(N) \\ &= -\left( N\gamma \left( 1-S\right) ^{2}+\gamma \left( \gamma +1-2S\right) \right) \\&\quad+\frac{N\left( \gamma -S^{2}\right) \left( \gamma -1\right) }{\gamma \left( N+1\right) ^{2}}\gamma \left( N+1\right) \\ &= -\frac{N^{2}\gamma \left( 1-S\right) ^{2}+N\left( \gamma \left( 1-S\right) \left( 3-S\right) +S^{2}\left( \gamma -1\right) \right) +\gamma \left( \gamma +1-2S\right) }{N+1} \\< & {} 0, \end{aligned}$$ which shows that $L(n)-R(n)$ is strictly decreasing.

### Comparative statics

Compute

$$\begin{aligned} \frac{d\lambda _{1}}{d\gamma } &= \frac{\left( N\left( 1-S\right) +1\right) ^{2}}{\gamma ^{2}\left( N+1\right) ^{2}}>0 \\ \frac{d\lambda _{1}}{dS} &= 2N\frac{1+N\left( 1-S\right) }{\gamma \left( N+1\right) ^{2}}>0, \end{aligned}$$

showing that the value of $\lambda _{1}$ increases with *S* and with $\gamma$, where $\gamma =\frac{F_{L}E_{H}^{2}}{F_{H}E_{L}^{2}}.$ If the ratio $\frac{E_{H}^{2}}{E_{L}^{2}}$ is kept constant, then $\lambda _{1}$ increases with $\frac{F_{L}}{F_{H}}$.

In the same way, compute

$$\begin{aligned} \frac{d\lambda _{2}}{d\gamma }&= \frac{\gamma +2NS\left( \gamma +N\left( \gamma -S\right) \right) }{\gamma ^{3}\left( N+1\right) ^{2}}>0 \\ \frac{d\lambda _{2}}{dS} &= -2N\frac{\gamma +N\left( \gamma -S\right) }{\gamma ^{2}\left( N+1\right) ^{2}}<0, \end{aligned}$$

so that the value of $\lambda _{2}$ decreases with *S* and increases with $\gamma$.

Finally, note that a change in $\delta$ has no impact on the function *L*, while an increase in $\delta$ shifts the function $R\left( n\right)$ upwards for all *n*; as a result, the intersection of the two functions happens at a smaller value of *n* (more *L* firms).

## Footnotes

1 https://www.nielsen.com/eu/en/insights/article/2018/global-consumers-seek-companies-that-care-about-environmental-issues/; https://www.forbes.com/sites/forbesnycouncil/2018/11/21/do-customers-really-care-about-your-environmental-impact/#6a24ad41240d; https://www.unilever.com/news/press-releases/2017/report-shows-a-third-of-consumers-prefer-sustainable-brands.html; https://www.independent.co.uk/life-style/food-and-drink/environment-meat-bottled-drinks-plastic-waste-consumer-customer-buy-spend-money-a9099446.html.equilibrium output of the oligopoly.

2 Given the result of firm’s entry on the total equilibrium quantities, the impact on the producers’ surplus is ambiguous.

3 For instance, to reduce the participation of green firms in the industry, the fixed cost needs to be at least 2.4% of the equilibrium profit in Fig. 9, whereas it needs to be at least 59% of the equilibrium profit in Fig. 11.
